# Gut microbiome drives individual memory variation in bumblebees

**DOI:** 10.1038/s41467-021-26833-4

**Published:** 2021-11-25

**Authors:** Li Li, Cwyn Solvi, Feng Zhang, Zhaoyang Qi, Lars Chittka, Wei Zhao

**Affiliations:** 1 State Key Laboratory of Food Science and Technology, School of Food Science and Technology, Jiangnan University, Wuxi, Jiangsu 214122 China; 2 Ecology and Genetics Research Unit, University of Oulu, Oulu, Finland; 3 School of Biological and Behavioural Sciences, Queen Mary University of London, London, E1 4NS UK; 4 Affiliated Hospital of Jiangnan University, Wuxi, Jiangsu 214122 China

**Keywords:** Long-term memory, Metagenomics, Microbiome

## Abstract

The potential of the gut microbiome as a driver of individual cognitive differences in natural populations of animals remains unexplored. Here, using metagenomic sequencing of individual bumblebee hindguts, we find a positive correlation between the abundance of *Lactobacillus* Firm-5 cluster and memory retention on a visual discrimination task. Supplementation with the Firm-5 species *Lactobacillus apis*, but not other non-Firm-5 bacterial species, enhances bees’ memory. Untargeted metabolomics after *L. apis* supplementation show increased LPA (14:0) glycerophospholipid in the haemolymph. Oral administration of the LPA increases long-term memory significantly. Based on our findings and metagenomic/metabolomic analyses, we propose a molecular pathway for this gut-brain interaction. Our results provide insights into proximate and ultimate causes of cognitive differences in natural bumblebee populations.

## Abstract (web summary)

Whether gut microbes drive cognitive differences in natural populations of animals remains unknown. Here, Li *et al*. demonstrate a causal link between increased symbiotic *Lactobacillus* Firm-5 species (*L. apis*) and improved long-term memory in bumblebees.

## Introduction

There is a growing interest in determining the causes of variation in individual cognitive abilities found within natural populations of animals^1,2^. Davidson et al.^3^ proposed that research on individual variation in the gut microbiome and cognition in natural populations will provide new insight into environmental and evolutionary factors that drive individual cognitive differences. Studies have revealed a major role for the microbial communities within the gut on brain function, e.g., gut microbes affect the level of neurotransmitters, the expression of neural receptors, synaptic plasticity and neurogenesis in the brain^4,5^. However, we do not yet understand the effects of gut microbiota on learning and memory in natural populations of non-human animals.

Bees’ cognitive abilities vary across individuals and they possess a relatively small community of gut microorganisms compared with mammals, making them ideal models to explore the role of specific gut symbiotic bacteria on individual cognitive variations in natural populations^6–10^. Similar to the human gut microbiota, bee gut bacteria are specific to the host gut. These bacteria can be transmitted from one individual to another via foraging on flowers and certain social interactions within the nest^7,11^. The bee hindgut (ileum and rectum) has the highest abundance of bacteria and is dominated by five core bacterial species clades (phylotypes *Snodgrassella alvi*, *Gilliamella apicola*, *Lactobacillus* Firm-4, *Lactobacillus* Firm-5, and *Bifidobacterium* species)^7^. Each bacterial species within this core group are thought to be symbiotic and possess distinct metabolic functions linked to mutualistic interactions with the host, as well as biofilm formation, and carbohydrate breakdown^7,12,13^.

Here, we set out to test whether individual cognitive variation across bumblebees might stem from microbiota-gut-brain interactions, and specifically ask which and how specific gut microbes might drive these cognitive differences in normal, healthy bumblebees. In this work, we identify a causal link between increased symbiotic *Lactobacillus* Firm-5 species (*L. apis*) and improved long-term memory in bumblebees.

## Results

### Long-term memory retention correlates with *Lactobacillus* Firm-5 abundance

**Fig. 1:**
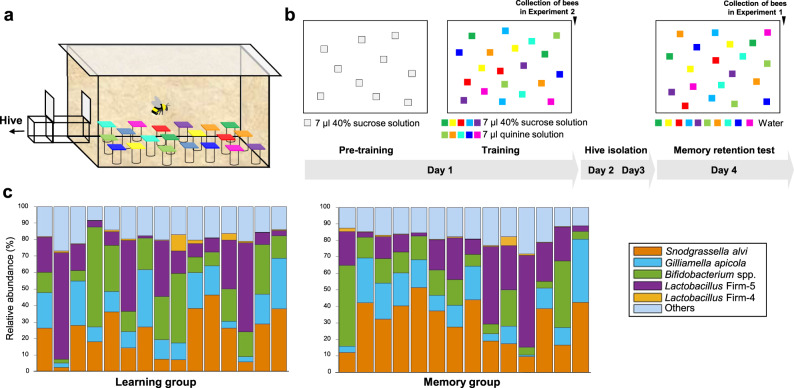
Behavioural paradigm and gut microbiome composition of individual bees. **a** Foraging arena setup with artificial flowers used for 10-Colour Learning. **b** Training procedures. Grey squares: transparent chips. Coloured squares: coloured chips (Supplementary Fig. 1a and the authors’ published work^9^). **c** The relative abundance of gut bacterial phylotypes in individual bees from the Learning group (*n* = 15) and Memory group (*n* = 14). Each column represents an individual bee. Source data are provided as a Source Data file.

First, we evaluated the learning speed and memory retention of individual bumblebees in a visual learning task (Supplementary Movies 1–3), and subsequently analysed the gut microbiome via metagenomic shotgun sequencing. Bees had to distinguish five different rewarding colours from five different colours associated with punishment (Methods; Fig. 1a, b and Supplementary Fig. 1a; Supplementary Movies 1–3). One group of bees was collected immediately after training (Learning group) and another group was collected immediately after a retention test 3 days after training (Memory group).

The gut microbiota of 29 hindgut samples (15 bees in the Learning group and 14 bees in the Memory group) were analysed by metagenomic sequencing, yielding an average of 80.99 million reads (10 Gb data) per sample. After quality control and removal of reads mapping to the bumblebee genome, the remaining microbiome reads (an average of 11.50 million reads per sample) were used to evaluate the abundance and function of the gut microorganisms (Supplementary Data 1). At the genus level, the five most dominant genera (*Snodgrassella*, *Lactobacillus*, *Gilliamella*, *Bifidobacterium* and *Candidatus Schmidhempelia*) constituted 92% of the microbiome on average (Supplementary Fig. 1b and Supplementary Data 2). There was also pronounced interindividual variation for bee gut microbiota composition (Supplementary Fig. 1b).

**Fig. 2:**
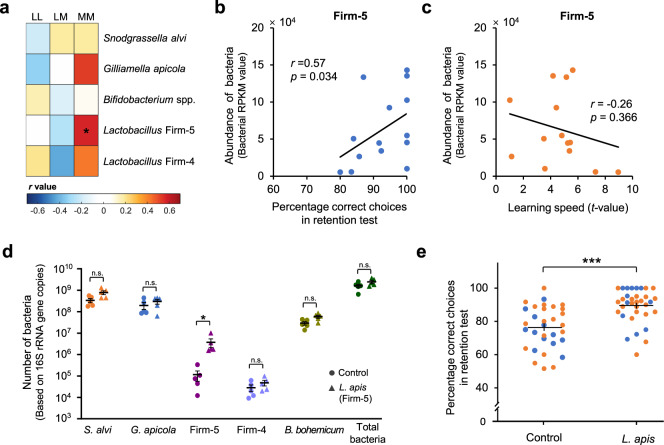
Individual long-term memory retention correlates with the abundance of bee gut microbes, and *L. apis* supplementation enhances long-term memory. **a** The correlations between learning speed/memory retention and the abundance of bacterial phylotypes. Colours indicate the coefficient *r* values from two-sided Spearman correlation analyses (*p = 0.034). Bonferroni corrected *p* values for all spearman correlations are shown in Supplementary Data 3. *LL* learning speed in the Learning group (*n* = 15 bees), *LM* learning speed in the Memory group (*n* = 14 bees), *MM* memory performance in the Memory group. **b** The percentage of correct choices in the memory retention test correlated positively with the abundance of *Lactobacillus* Firm-5. **c** Learning speed did not correlate with Firm-5 abundance. The *t* value is the indicator for learning speed (Methods). High *t* values indicate slow learning whereas low *t* values indicate fast learning. Coefficient *r* and significance *p* values in (**b**) and (**c**) are from Spearman correlation analyses, *n* = 14 bees. **d** *L. apis* supplemented diet increased its abundance in the bee hindgut (two-sided Mann-Whitney *U* test with Bonferroni correction: Firm-5, *U* = 0, *n* = 5 bees for both groups, *p* = 0.048) compared with Control (sugar without *L. apis*). **e** Bees with *L. apis* increase in gut by supplementation (*n* = 34) had better long-term memory, compared with Control (*n* = 32) (GLMM, *df* = 64, ***p = 3.656 × 10^−5^; Supplementary Table 1). Filled circle colours indicate different colonies (*n* = 2). Data are presented as mean ± SEM in (**d**) and (**e**). Source data are provided as a Source Data file.

To identify the gut microbes which might play a role in individual cognitive differences, correlation analyses were performed between learning/memory performances and the five genera with the greatest abundance. For the Memory group, memory retention positively correlated with genus *Lactobacillus* (Spearman’s *r* = 0.54, *n* = 14, *p* = 0.047; See Supplementary Data 3 for Bonferroni corrected *p* values for all spearman correlations; Methods). This correlation was validated with a partial correlation analysis between memory retention and the five genera (*n* = 14, *r* = 0.74, *p* = 0.014). Memory performance did not correlate with the abundance of any other genus (Supplementary Data 3). For both the Learning and the Memory groups, learning speed (assessed based on landings on flowers during training) did not correlate with the abundance of any genus (Supplementary Data 3). Because the genus *Lactobacillus* includes the two dominant phylotypes *Lactobacillus* Firm-4 and Firm-5 in bee guts, the gut microbes were then analysed at the phylotype level to find out which *Lactobacillus* group may affect memory. There were five main phylotypes (*Snodgrassella alvi*, *Gilliamella apicola*, *Bifidobacterium spp*., *Lactobacillus* Firm-4 and Firm-5) with an average relative abundance higher than 1%, and their total abundances accounted for 82% of the microbiome on average (Fig. 1c and Supplementary Data 2). A positive correlation was only found between memory retention and the abundance of *Lactobacillus* Firm-5 (Fig. 2a, b; Spearman’s *r* = 0.57, *n* = 14, *p* = 0.034). Memory retention did not correlate with the abundance of any other phylotype (Fig. 2a and Supplementary Data 3). The learning speed in the Learning and the Memory groups did not correlate with the abundance of any phylotype of bacteria (Fig. 2a, c and Supplementary Data 3). Quantitative real-time PCR determined the absolute abundance of the total bacteria in bee hindguts. The total abundance of all bacteria did not correlate with learning speed or memory for either the Learning or the Memory groups (learning speed in the Learning group: Spearman’s *r* = −0.04, *n* = 15, *p* = 0.883; learning speed in the Memory group: Spearman’s *r* = −0.16, *n* = 14, *p* = 0.573; memory performance in the Memory group: Spearman’s *r* = 0.26, *n* = 14, *p* = 0.372; Supplementary Fig. 2a, b).

### Increased gut *Lactobacillus apis* causes better long-term memory retention

The correlations found with the Memory group might have arisen from other factors (e.g., the genetic background or pre-adult development of the host), provoking the question of whether there is a causal link between the *Lactobacillus* Firm-5 cluster and the host’s long-term memory. To determine whether the natural variations in *Lactobacillus* Firm-5 caused the observed individual memory differences, we provided bees with a diet (sugar solution) containing the *Lactobacillus* Firm-5 species *L. apis*, thereby increasing the abundance of *L. apis* in the gut. We subsequently measured bees' learning speed and memory in the same foraging task. We chose *L. apis* because it was the most classified *Lactobacillus* species isolated from our bee gut samples on MRS agar (4 of 20 isolated strains were *L. apis*, most of the isolated strains were unclassified *Lactobacillus*). Further, metagenomic results showed that the abundance of genes mapped to *L. apis* was the highest among the Firm-5 species (Supplementary Fig. 1d), indicating that *L. apis* might be the dominant species in the Firm-5 community of our bumblebees. Supplementation of *L. apis* increased its abundance in the bee hindgut (Fig. 2d). Bees with increases in gut *L. apis* abundance by supplementation displayed better long-term memory retention (GLMM, *df* = 64, *p* = 3.656 × 10^−5^; Fig. 2e and Supplementary Table 1), but not learning speed (Supplementary Table 1), compared with control bees. To verify whether the observed memory enhancement was not a general response to increase of any bacterial species supplemented, two other groups of bees were given diets containing *S. alvi* or *G. apicola*, two dominant bacteria (Fig. 1c) that did not correlate with memory performance (Fig. 2a and Supplementary Data 3). Supplementation of *S. alvi* and *G. apicola* increased their abundance in the bee hindgut (Supplementary Fig. 2c; for *S. alvi*, the increase was significant, while for *G. apicola*, an increase was observed but not significantly (*p* = 0.056). Learning speeds and memory performances of bees with increases in *S. alvi* or *G. apicola* by supplementation were no different to the control group (Supplementary Fig. 2d and Supplementary Table 2).

### *Lactobacillus apis* supplementation promotes glycerophospholipid metabolism

Having established a causal link between natural variations of *L. apis* and individual memory retention, we aimed to explore the underlying mechanisms involved. One way to do this is by deciphering the functions encoded by bacterial DNA. Genes harboured by Firm-5 were compared with those harboured by *S. alvi* and *G. apicola* (KEGG (Kyoto Encyclopaedia of Genes and Genomes) pathways detected, Supplementary Data 4). The most abundant pathways in all three types of bacteria included Biosynthesis of amino acids, ABC transporters, Ribosome, Carbon metabolism, Pyrimidine metabolism and Purine metabolism, which were essential for bacteria survival. Comparing with *S. alvi* and *G. apicola*, Firm-5 specifically had a higher abundance of phosphotransferase system (PTS), Fructose and mannose metabolism and starch and sucrose metabolism pathways, which may be essential for Firm-5 to enhance memory.

The correlations between physiological indexes and the abundance of functional terms have been conducted before to speculate which function of the bacteria may cause the host physiological and behaviour changes^14,15^. We therefore examined the correlations between the abundance of functional terms (KEGG pathways, calculated based on Firm-5 genes) and long-term memory retention (Supplementary Data 5). We hypothesised that metabolites produced by these pathways might play a role in *L. apis*-induced memory enhancement. Of those pathways found to correlate with memory performance, the 20 most abundant are shown in Supplementary Fig. 3a (Firm-5). Because metabolites produced by the other gut bacteria may also help contribute to the effect on host cognition, we also assessed the correlations between the abundance of KEGG pathways associated with the entire gut microbiota and long-term memory performance (Supplementary Fig. 3a (Gut Microbiota) and Supplementary Data 5). Several pathways were found in both analyses to correlate with long-term memory performance, including Starch and sucrose metabolism, Glycolysis/Gluconeogenesis, Glycerophospholipid metabolism and Purine metabolism (Supplementary Fig. 3a), suggesting that these pathways may contribute to the *L. apis*-induced memory enhancement.

**Fig. 3:**
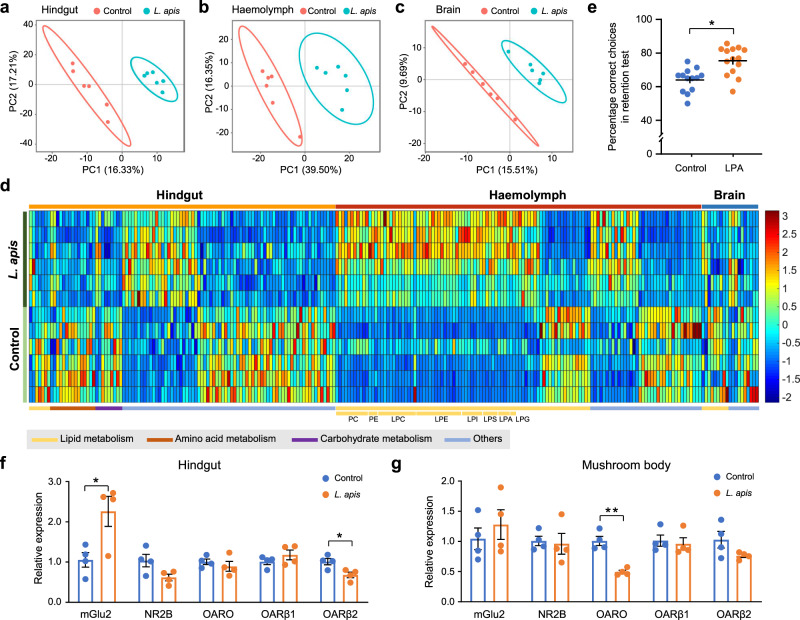
*L. apis* supplementation affects host metabolites and neural receptor gene expressions in bees. **a**–**c** Metabolomic analyses of bumblebee hindgut (**a**), haemolymph (**b**) and brain (**c**) samples from control and *L. apis-*fed bees. Partial least-squares discrimination analysis (PLS-DA) showed that the metabolomic profiles of the hindgut, haemolymph and brain in *L. apis*-fed and control bees differed (*n* = 6 for both groups). **d** Heatmap showing significantly altered metabolites in the bumblebee hindgut, haemolymph and brain. Colours indicate the normalised abundance of each metabolite. **e** The effect of one glycerophospholipid LPA (14:0) on long-term memory (GLMM, df = 24, *p* = 0.028; *n* = 13 for the Control group and *n* = 14 for the LPA group; Supplementary Table 3). **f** and **g** *L. apis* supplementation affects the gene expression of neural receptors in the host hindgut and brain mushroom body (two-sided Student’s *t* test: mGlu2 in the hindgut, *t*_6_ = 2.9175, *p* = 0.027; OARβ2 in the hindgut, *t*_6_ = −3.1301, *p* = 0.020; OARO in the mushroom body, *t*_6_ = −6.3565, *p* = 7.110 × 10^−4^; *n* = 4 bees for both groups). Asterisks indicate significant differences (*p < 0.05, **p < 0.01). Data are presented as mean ± SEM. *PC* phosphatidylcholine, *PE* phosphatidylethanolamine, *LPC* lysophosphatidylcholine, *LPE* lysophosphatidylethanolamine, *LPI* lysophosphatidylinositol, *LPS* lysophosphatidylserine, *LPA* lysophosphatidic acid, *LPG* lysophosphatidylglycerol, *mGlu2* metabotropic glutamate receptor 2, *NR2B* glutamate receptor ionotropic NMDA 2B, *OARO* octopamine receptor Oamb, *OARβ1* Octopamine receptor beta-1R, *OARβ2* Octopamine receptor beta-2R. Source data are provided as a Source Data file.

Another way of looking at the potential mechanisms underlying the observed gut-brain communication is by investigating abundance changes in metabolites further downstream (i.e., towards the brain). We performed untargeted metabolomics to determine metabolites potentially involved in *L. apis*-caused memory enhancement. Partial least-squares discrimination analysis (PLS-DA) showed that the metabolomic profiles of hindgut, haemolymph (fluid analogous to blood) and brain samples from *L. apis*-treated bees were different from those of control bees (Fig. 3a–c). The metabolites with abundance changes in the hindgut, hemolymph, and brain are shown in Supplementary Data 6. Within the haemolymph, the abundance of 122 metabolites was found to be significantly different between the two groups. Fifty-nine (48%) of these were glycerophospholipids, including phosphatidylcholines (PCs), phosphatidylethanolamines (PEs), and lyso-variants lysophosphatidylcholines (LPCs), lysophosphatidylethanolamines (LPEs) and lysophosphatidylinositols (LPIs) (Fig. 3d and Supplementary Data 6). Fifty-eight of the 59 glycerophospholipids increased in the haemolymph of bees treated with *L. apis*. Although most of these 58 metabolites were found in both hindgut and brain, *L. apis* supplementation did not alter their abundance in these two tissues (Supplementary Fig. 4a; Supplementary Data 7). Maltose, maltotetraose, maltopentaose and d-fructose 6-phosphate increased significantly while d-glycerate decreased significantly in the hindgut of bees supplemented with *L. apis*, which is related to sugar metabolism and glycerophospholipid metabolism (Supplementary Data 6). Several other glycerophospholipids decreased in the brains of bees treated with *L. apis*, including the phosphatidic acid PA(18:1/18:3), the phosphatidylcholines PC(18:5e/20:5), PC(16:2e/22:6) and PC(5:0/13:1), the lysophosphatidylcholine LPC(20:0) and the lysophosphatidylserine LPS(16:0) (Supplementary Data 6).

To validate the impact of the upregulated glycerophospholipids induced by *L. apis* supplementation on memory performance, Lysophosphatidic acid LPA(14:0), which increased the most in the haemolymph after *L. apis* treatment, was supplemented into bee food (sugar solution). LPA administration increased long-term memory significantly (GLMM, *df* = 24, *p* = 0.028; Fig. 3e and Supplementary Table 3). These results suggest that the underlying mechanism for the observed *L. apis-*induced memory enhancement may rely on increased glycerophospholipids.

### *L. apis* supplementation affects the expression of neural receptors in the hindgut and brain

Our results so far indicate that increased abundance of *L. apis* in the bumblebee gut leads to increased glycerophospholipid metabolism in the gut and increased glycerophospholipids in the haemolymph. To help gain a broader picture of the pathway between *L. apis* and memory enhancement, we examined the expression patterns of neural receptors (glutamate, dopamine, octopamine, acetylcholine and 5-HT) and neural synapsin (13 genes in total) in the hindgut and the brain. Within the brain, we looked particularly at the mushroom bodies, which are high-level sensory integration centres involved in learning and memory^16^. In the hindgut, *L. apis* supplementation increased the expression of the gene mGlu2 (Metabotropic glutamate receptor 2) (Fig. 3f; *t* test: *t*_6_ = 2.9175, *p* = 0.027), and decreased the expression of OARβ2 (octopamine receptor beta-2R) (Fig. 3f; *t* test: *t*_6_ = −3.1301, *p* = 0.020). In the mushroom bodies, *L. apis* supplementation decreased the expression of OARO (Octopamine receptor Oamb) (Fig. 3g; *t* test: *t*_6_ = −6.3565, *p* = 7.110 × 10^−4^), but did not affect the expression patterns of any other neural receptors in the hindgut or mushroom bodies (Fig. 3f, g and Supplementary Fig. 4b, c).

## Discussion

We found that variations in *L. apis* abundance in the gut cause individual memory differences in bumblebees. Our metagenomic results showed that a high abundance of sugar metabolism pathways were observed in Firm-5, and the glycerophospholipid metabolism pathway and several sugar metabolism pathways correlated with memory performance (Supplementary Fig. 3a, b). The metabolomic results indicate that more *L. apis* in the hindgut resulted in increased glycerophospholipids abundance in the haemolymph. Further, dietary supplementation of one of the increased glycerophospholipids LPA (14:0) enhanced long-term memory significantly, revealing that *L. apis* enhanced memory via glycerophospholipid metabolism.

For the gut microbes-memory correlations observed in our study based on the metagenomic and behavioral experiments, it may be that the correlations are a result of pre-adult (e.g., larvae) developmental differences or other factors, which lead to both memory differences and gut microbiome differences in the adult^17,18^. However, dietary supplementation of *L. apis* enhanced bees’ memories. These results suggest that the adult microbiome directly affects memory abilities in the adult.

**Fig. 4:**
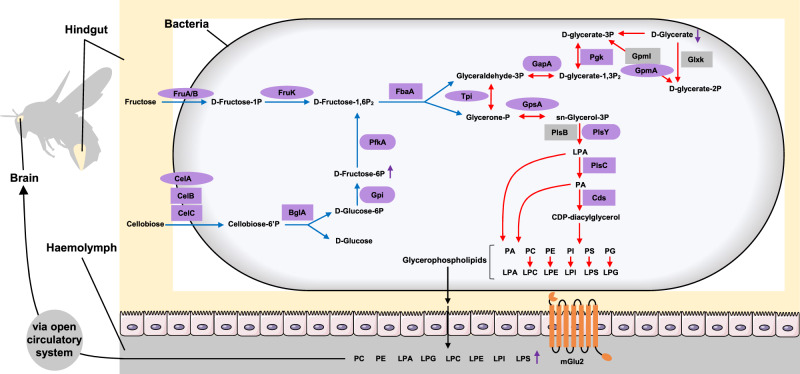
Proposed pathway between intestinal glycerophospholipid production and memory enhancement. Based on our metagenomic and metabolomic analyses, we suggest that increased levels of *L. apis* (and other Firm-5 species which similarly contain many genes for the PTS sugar transport pathway) will cause increased sugar metabolism, which will lead to the production of more glycerophospholipids. These glycerophospholipids are then transported into the hindgut and are rapidly secreted into the haemolymph (perhaps helped by an increase in mGlu2 receptors). They are transported to the brain via the bee’s open circulatory system, with attached metabolites, leading to improved structure and function of neural and synaptic membranes, thereby promoting better memory. Purple shapes: genes that positively correlated with long-term memory; rectangles: genes found in all three analyses (analyses based on genes mapped to the whole gut microbiota, Firm-5 and *L. apis*); Ovals: genes found in whole gut microbiota and Firm-5 analyses; Rounded rectangles: remaining memory-correlated genes; Grey shapes: genes which did not correlate with long-term memory; Blue arrows: sugar metabolism; Red arrows: glycerophospholipid metabolism; Purple arrows: the increase or decrease of metabolites after *L. apis* supplementation.

The metabolites found to change in the hindgut as a result of increased *L. apis* are known to play roles in lipid, amino acid, and carbohydrate metabolism (Fig. 3d). Together with the dominant pathways in Firm-5, the memory-correlated pathways, the sugar and glycerophospholipid metabolic genes that correlated with memory performance (Supplementary Data 8; Supplementary Figs. 5–8), we propose a potential molecular pathway for how increased *L. apis* abundance in the hindgut leads to increased abundance of glycerophospholipids in the haemolymph and eventually to enhanced memory (Fig. 4). Genome analyses have confirmed the presence of an exceptionally large number of PTS (a distinct pathway used by bacteria for sugar uptake) genes for the Firm-5 species^19^. Our result showed that Firm-5 particularly had a higher abundance of PTS pathways compared with that in *S. alvi* and *G. apicola*. We therefore suggest that in bumblebees, increased abundance of *L. apis* (along with the other Firm-5 species) will increase uptake of fructose and cellobiose (based on the memory-correlated genes) present in the gut as a result of a normal diet and convert them to d-Fructose-1,6P_2_ which is then broken down into glyceraldehyde-3P and glycerone-P. As essential components of glycerophospholipid metabolism, the higher abundance of glyceraldehyde-3P and glycerone-P will then lead to higher production of glycerophospholipids in the bacteria. While all of the dominant bacteria have genes for glycerophospholipid metabolism. It is likely that Firm-5 can produce some essential intermediate metabolites from the sugar metabolism, which can be used by Firm-5 and other bacteria to generate more glycerophospholipids. These glycerophospholipids will subsequently be transported into the hindgut^20^, and from there secreted into the haemolymph^21^. We did find that *L. apis* supplementation increased the expression of the gene mGlu2 in the hindgut. It may be that increased mGlu2 receptors, which have been found to promote membrane secretion and motility^22^, help rapidly transport the glycerophospholipids from the hindgut into the haemolymph (Fig. 4). Whether and exactly how the observed changes in octopamine receptors’ gene expressions are involved in the memory-enhancing effects of *L. apis* will require further investigation. In addition, the changed metabolites in the hindgut may be used directly by the host to produce glycerophospholipids or just simple stimulators that induce the host to do so. Our results suggest that the observed *L. apis*-induced memory enhancement is due at least in part to increased glycerophospholipid abundance in the haemolymph.

Glycerophospholipids are the major constituent of neural membranes and are vital for the integrity, stability, permeability, and fluidity of these membranes. Low levels of these phospholipids have been linked to disorders with memory impairment^23,24^. Glycerophospholipid supplementation has been found to improve cognitive function, reduce factors linked to cognitive decline, and benefit cerebral structure in humans, rats and mice^21,24^. Glycerophospholipid degradation can also produce second messengers such as diacylglycerol and arachidonic acid, which are important for synaptic plasticity and cognition^25,26^.

If our surmised molecular pathway is correct, how are the glycerophospholipids getting to the brain? Gut microbiota produces numerous metabolites that may be transported to and accumulate in the mammalian blood where they are transported to various parts of the host’s body and affect physiology and behaviour^27,28^. We suspect that increased levels of *L. apis* in the gut result in a larger pool of glycerophospholipids in the haemolymph, and that these metabolites are naturally transported via their open circulatory system to and used by the brain. The glycerophospholipids found to have less abundance within the brains of *L. apis*-fed bees carry essential polyunsaturated fatty acids (e.g., α-linolenic acid, ALA, 18:3; docosahexaenoic acid, DHA, 22:6; eicosapentaenoic acid, EPA, 20:5) which are known to benefit learning and memory by supporting neural membrane structure, function and plasticity in the brain^29–31^. Therefore, we speculate that polyunsaturated fatty acids transported to the brain via glycerophospholipids (which are catabolized to release these fatty acids, leading to the decrease of the glycerophospholipids) may be a key contributing factor in the observed *L. apis*-caused memory differences in bumblebees. The pathway we proposed is the most likely one based on our findings. However, other pathways (e.g., the amino acid metabolism pathway and immune pathways) may also play important roles in this gut-brain interaction.

Variations in the microbiome across individual bumblebees can arise from fluctuations in nest environment, activities, pathogens, social interactions, and pollination environment^7,11,32^. One possibility that could be derived from our findings is that memory enhancement might be a sort of currency paid by the bacteria as part of their symbiosis with the bees. It has been suggested that gut bacteria may shape the physiological ability of queens to survive^33^. Bacteria that can promote cognitive ability in bumblebee workers theoretically should have a better chance at increasing their own fitness. Bumblebee workers with better cognitive abilities should survive longer and bring back more food to the colony^34^, thereby increasing the number of bacteria deposited in the nest and to nestmates. As a result, the bacteria then have a greater chance of being taken up by the virgin queens in the later stages of the colony’s life, and increase their chances of being passed on to the next generation of bumblebees^35^. Moreover, researchers have suggested that the effect of gut bacteria on host behaviour is a by-product of natural selection on microorganisms to grow within the host^36^. A further consideration is that the memory enhancement might be directed towards flowers from which bumblebees might obtain the memory-enhancing bacteria^3,37^. This of course would require that these bacteria thrive only in certain types of flowers, but certain types of bacteria, including *Lactobacillus* Firm-5, have been detected at much higher levels in certain species of flowers^38^.

Here, we show that variation in a symbiotic bacterial species in the gut causes better long-term memory on a visual-discrimination foraging task in bumblebees. Our results suggest that underlying mechanisms may involve gut bacteria-induced glycerophospholipids, which accumulate in the haemolymph and, we speculate, may benefit brain structure and function and thereby improve memory capacities. These findings provide valuable information on a natural driver of individual cognitive variation.

## Methods

### Animals

Bumblebee colonies (*Bombus terrestris*) were purchased from Koppert B.V. (Beijing, China). All colonies were settled in wooden nest boxes (40 × 28 × 11 cm), which were connected to small flight arenas (65 × 45 × 25 cm) through a Perspex corridor (25 × 3.5 × 3.5 cm). There were small doors in the corridor which allowed us to control when and which bees were able to enter the arena. Bee identity was tracked with individual number tags (Opalithplättchen, Warnholz & Bienenvoigt, Ellerau, Germany) glued to the top of the bee thorax by means of Superglue. Illumination in the lab was controlled with a 12 h day–night cycle (8:00 am–8:00 pm). Bees had no foraging experience in the arena prior to pretraining. All bees used in the experiments were worker bees and had similar ages (12.18 ± 0.52 days) at the time of collection.

#### Animal welfare

Although there are no current requirements regarding insect care and use in research, our experimental design and procedures were guided by the 3Rs principles^39^. The bumblebees were cared for on a daily basis by trained and competent staff, which included routine monitoring of welfare and provision of correct and adequate food (pollen and sucrose solution) during the experimental period. Bees voluntarily foraged in behavioural experiments. The behavioural tests were non-invasive and the types of manipulations used (sucrose, bitter substances) are all experienced by bumblebees during their foraging life in the wild. For the molecular analyses, bees were collected gently with forceps and holding them above dry ice for several seconds immediately after collection until completely anaesthetised from the sublimated CO_2_. This quick process was done to reduce any unnecessary stress on the bees as much as possible.

### Behavioural assay

We used a behavioural assay, a 10-colour learning paradigm, established in our earlier work^9^, which is difficult enough to show large performance variations across individuals.

#### Pretraining

All bees were first trained to land on transparent Perspex chips (25 × 25 mm; artificial flowers—henceforth, “flowers”) with 7 μl 40% sucrose solution. Five flowers were arranged in a pseudorandom array within the arena (Fig. 1a), each on top of a small glass vial. Bees successfully foraging from the transparent chips and returning to the colony 8–10 times regularly (inter-trip-interval within 5 min) were regarded as good foragers and moved on to the training phase.

#### Training

Bees were trained individually to discriminate five different flowers containing sucrose solution from five different flowers containing bitter quinine solution (Fig. 1a, b). Bees had five foraging trips with 10 min inter-trip-intervals, which can cause long-term memory formation in bees. Flowers were cleaned with 70% ethanol in water between trips to ensure no scent marks were used to solve the task. The ten flower colours used here were the same as before and bees showed no preference for any rewarding colours (Supplementary Fig. 1a and our published work^9^). There were a total of 20 artificial flowers in the arena with two flowers for each colour. All rewarding flowers contained 7 μl 40% sucrose solution, and all unrewarding flowers contained 7 μl saturated quinine solution (1.2 mg/ml H_2_O). All landings to flowers in the training were recorded to evaluate learning speed. A landing was defined as any time the bee was positioned on top of a chip and they stopped flying (wing movements ceased) for any amount of time. After training, bees were confined to the nest for three days to prevent any further foraging experience. During this time, the colony was fed with 40% sucrose solution pipetted directly into their cells every day (~10 ml). On day four, bees received a memory retention test on the same flower setting as in the training, except that each flower contained 7 μl water. All landings to flowers within three minutes of entering the arena were recorded to evaluate memory retention. For the Learning group, age-matched bees (*n* = 15, 12.33 ± 0.49 days) were collected immediately after the final trip of the training on the first day for gut microbiota determination, thereby allowing us to investigate the relationships between learning speed and the gut microbiota. For the Memory group, age-matched bees (*n* = 14, 12.50 ± 0.65 days) were collected immediately after the retention test for gut microbiota determination, thereby allowing us to investigate the relationships between memory retention and gut microbiota. Bees in both groups were from the same colony.

### Metagenomic analysis of the hindgut microbiota

The hindguts of the bees from the Learning and Memory groups were dissected out by sterile fine-tipped forceps. Total genomic DNA was extracted from each bee hindgut (29 samples in total) by using the E.Z.N.A. Stool DNA Kit (Omega Bio-tek, Norcross, GA, USA) according to the manufacturer’s instructions. Illumina paired-end libraries were generated using NovaSeq 6000 S4 Reagent Kit (Illumina, San Diego, CA, USA). Following this, the library was paired-end sequenced on the Illumina NovaSeq^TM^ 6000 Sequencing System with read length of 150 bp at HonSunBio Co., Ltd. (Shanghai, China). More than 10 Gb data (on average 80.99 million reads) were generated per sample.

The low-quality reads were removed by Sickle (https://github.com/najoshi/sickle) (-q 20 -l 50) and the reads mapping to the bumblebee (*Bombus terrestris*) genome (Bter_1.0) by BWA (http://bio-bwa.sourceforge.net) were also filtered out. The high-quality sequencing reads were used for assembling contigs by MEGAHIT (Version 1.1.2)^40^ (-k 47:97 -minLen 300), and contigs with the length ≥300 bp were used for the following analyses. MetaGene was applied for open reading frame prediction of the assembled contigs^41^. All predicted protein-coding genes with a threshold of 95% sequence identity and 90% coverage were clustered as the non-redundant gene catalogue (101,659 non-redundant genes) by CD-HIT (Version 4.6.1)^42^ (-c 0.95 -a 0.9). The abundance of each gene was quantified with SOAPaligner (Version 2.22) (-r 1 -l 35 -M 4 -S -p 6 -v 20 -c 0.95) by aligning high-quality sequencing reads to the non-redundant gene catalogue with 95% identity^43^. The amino acid sequence of each non-redundant gene was blasted against NCBI NR database for taxonomic annotation and blasted against the KEGG database for functional annotation with *e* value <1e^−5^ by DIAMOND (Version 0.8.35)^44^. Genes were assigned to the microbial taxa with the highest scores, and the total read count of all assigned genes to a specific taxon was used to estimate its abundance. The normalised read counts RPKM (Reads Per Kilobase of per Million mapped reads) values of taxa or KEGG terms were used for statistical analyses^15^. The relative abundance of a taxon was calculated by dividing its RPKM with the total RPKM of all taxa in one sample. The primary annotation data are shown in Supplementary Data 9. Permutational multivariate analysis of variance (PERMANOVA) was conducted to compare the microbiota composition between the Learning group and Memory group, according to the genera that were shared by >20% of the samples. The gut microbiota composition of the Learning group did not differ from the Memory group (Supplementary Fig. 1c; PERMANOVA, *R*^2^ = 0.0364, *p* = 0.378), which suggests that the learning and memory states (or collection time after training) did not affect bee gut microbiota structure.

The housekeeping genes of each of the four most abundant species were analysed to determine whether we could confidently identify bacteria at the species level with the gene-mapping method we conducted, described above. The core genes of each species based on our gene catalogue were extracted and compared with those extracted from the reference genome using the up-to-date bacterial core gene (UBCG) pipeline (Version 3.0)^45^. The sequence of each core gene was aligned with the reference core genes (92 core genes for each species) individually, and the nucleotide identity was calculated for each gene (Supplementary Data 10). Some studies have used 31 phylogenetic marker genes to identify species^46^. However, not all the 31 housekeeping genes were detected and with >95% sequence identity (Supplementary Data 10). Therefore, we cannot be confident in the species assignments using the gene-mapping approach. We then performed three other analyses (mOTU, MetaPhlAn3, Kraken2) in attempts to identify bacteria at the species level. However, we were again not confident in any of these species assignment results (see Supplementary Methods for details; Supplementary Data 11). Therefore, we presented and analysed our microbial data at the level of phylotypes.

### Diet supplementation with *Lactobacillus apis* and other gut bacteria

*Lactobacillus apis* was isolated from the hindguts of bumblebees (*Bombus terrestris*) using MRS agar, and *Snodgrassella alvi* and *Gilliamella apicola* were isolated from the bumblebee hindguts with standard LB (lysogeny broth) agar as previously described^47,48^. We name these strains *Lactobacillus apis* BB1*, Snodgrassella alvi* BB3, and *Gilliamella apicola* BB10. These strains were identified by the 16 S rRNA gene sequencing (the similarity of these sequences to the closest strain sequence: *L. apis*, 99.73% to that of strain ESL0185, CP029476.1; *S. alvi*, 99.15% to that of strain R-53583, LT631744.1; *G. apicola*, 99.09% to that of strain wkB30, JQ936676.2). *L. apis BB1* was grown in MRS broth at 37 °C under anaerobic conditions. *S. alvi BB3* and *G. apicola BB10* were grown in LB medium at 37 °C under a CO_2_-enriched atmosphere (6%) for 48 h in 12-ml tubes. After 2 days, bacteria cultures were centrifuged at 8000 × *g* for 1 min, washed three times with sterile PBS, and diluted in 40% sucrose solution to OD_600_ = 1.0. Bees that were three days old (from emergence) were moved into small wooden boxes and provided with adequate pollen and fresh sucrose solution (with *L. apis* or *S. alvi* or *G. apicola*, or without gut bacteria), which was made daily and supplied to different treatment groups of bees between 15:00 and 16:00 for 9 days. For the experiment to validate the effect of specific gut bacteria on long-term memory performance, the bees were moved back into their colony for behavioural training (learning assay) on day 6 and were put back into the small wooden boxes after training. On day 9, bees were placed back into their colony again for the memory retention test and were collected immediately after the test (bees were anaesthetised with CO_2_ by holding them above dry ice for several seconds). The hindgut was dissected out and the haemolymph was collected from an incision at the base of the head of each bee immediately after sample collection. Two independent experiments (two colonies) were conducted to examine the effect of *L. apis* supplementation on long-term memory (Colony 1: control *n* = 12, *L. apis n* = 12; Colony 2: control *n* = 20, *L. apis n* = 22;). One new colony was used to examine the effect of *S. alvi* and *G. apicola* supplementation on long-term memory (Colony 3: control *n* = 16, *S. alvi n* = 18, *G. apicola n* = 17).

### Metabolomic analysis of the hindgut, haemolymph and brain samples

A total of 147 bees from two colonies were used for the untargeted metabolomic analysis. For each group, bees were collected from both colonies evenly. The *L. apis* supplementation group consisted of 35 bees from colony 1 and 40 bees from colony 2. The control group consisted of 37 bees from colony 1 and 35 bees from colony 2. The hindguts, haemolymph or brains from 11 to 14 bees (the same colony) were pooled in each sample and there were six biological replicates for each group. Metabolites were extracted from each sample, and liquid chromatography–mass spectrometry (LC–MS) analyses were performed using a Vanquish UHPLC system (Thermo Fisher) coupled with an Orbitrap Q Exactive HF-X mass spectrometer (Thermo Fisher) in both positive and negative ionisation modes by Novogene Co., Ltd. (Beijing, China). Three pipelines were conducted for the three different types of tissues separately.

#### Metabolites extraction

Tissues in each sample were grounded and homogenised in liquid nitrogen, and they were added to prechilled 80% methanol with 0.1% formic acid (500 μl for 100 mg gut and brain tissues, 400 μl for 100 μl haemolymph). After vortexing, the samples were incubated on ice for 5 min and were centrifuged at 15,000 × *g*, 4 °C for 5 min. The supernatant was subsequently diluted in LC–MS grade water to the final concentration of methanol was 53%, which were subsequently transferred to a new Eppendorf tube and then were centrifuged at 15,000 × *g*, 4 °C for 10 min. Finally, the supernatant was injected into the LC–MS/MS system. Same amounts of supernatant from each treated sample within one pipeline were pooled and used as a QC (quality control) sample.

#### UHPLC-MS/MS analysis

LC separation was performed by Hyperil Gold column (100 × 2.1 mm, 1.9 μm) using a 16-min linear gradient at a flow rate of 0.2 ml/min. The eluents for the positive mode were eluent A (0.1% formic acid in water) and eluent B (methanol). The eluents for the negative mode were eluent A (5 mM ammonium acetate, pH 9.0) and eluent B (methanol). The gradient programme was set as follows: 0–1.5 min, 2% B; 12.0 min, 2–100% B; 14.0 min, 100% B; 14.1 min, 100–2% B; 17 min, 2% B. The Q Exactive HF-X mass spectrometer was operated in positive and negative polarity mode with a spray voltage of 3.2 kV, capillary temperature of 320 °C, sheath gas flow rate of 35 arb and aux gas flow rate of 10 arb. The data were acquired with the Xcalibur 4.1 (Thermo Fisher) and Tune 2.9 (Thermo Fisher).

#### Data analysis

To identify and quantify the metabolites, the raw data files were processed and matched against mzCloud (https://www.mzcloud.org/) and the local databases mzVault and MassList using the Compound Discoverer 3.1 (CD3.1, Thermo Fisher). For functional annotation, these metabolites were mapped to the KEGG database, HMDB database and LIPID MAPS database. PLS-DA was conducted to visualise the discrimination between the *L. apis* supplementation and Control groups. The metabolites with Variable Importance in Projection (VIP) > 1, and *p* value (two-sided *t* test) < 0.05 were defined as differentially expressed metabolites.

### Oral administration of LPA

Lysophosphatidic acid LPA(14:0) (Avanti Polar Lipids, 857120 P) was dissolved in 40% sucrose solution with 0.1% bovine serum albumin (BSA) to 300 μM. Bees with an age of 3 days were moved into small wooden boxes and provided with adequate pollen and sucrose solution containing LPA (LPA group) for nine days. For the control group, sucrose solution with 0.1% BSA did not contain LPA. On day 6, bees were placed back into their colony for behavioural training (learning assay) and on day 9, bees were placed back into their colony again for a memory retention test.

### DNA/RNA isolation and quantitative real-time PCR

The absolute quantity of total gut bacteria and specific members of the gut microbiota was determined with quantitative PCR following the method described by Kešnerová et al.^12^. The genomic DNA was extracted from each bee hindgut by using the E.Z.N.A. Stool DNA Kit (Omega Bio-tek). Quantitative PCR was performed on the QuantStudio 3 (Applied Biosystems) using the iTaqTM Universal SYBR Green Supermix (Bio-Rad) in a 96-well plate (10-μl reactions) according to the manufacturer’s protocol. Standard curves of plasmids containing the target sequence (species-specific 16 S rRNA gene sequence and universal 16 S rRNA gene sequence) were generated for absolute quantification. Serial dilutions with the final concentrations of the plasmid 10^8^ to 10^2^ copies per μl were used. The absolute bacterial 16 S rRNA gene copies of a specific species or universal bacteria in DNA samples extracted from bee hindguts were calculated based on the standard curve. Finally, the bacterial 16 S rRNA gene copies were normalised to the medium number of host actin gene copies to reduce the effect of gut size and DNA extraction efficiency in the qPCR experiments. Universal and specific bacteria primers are shown in Supplementary Table 4.

Whole brains were dissected out over ice/dry ice mixture and small pieces of hair or trachea were removed by fine-tipped forceps. On the ice/dry ice mixture, the structure of the mushroom body of the brain was easily discernible under the dissecting microscope. The mushroom body was separated along its edge by a fine scalpel^49^, and then stored in the centrifuge tube. The mushroom body or the hindgut from two bees were pooled into one sample for RNA extraction. Total RNA was extracted from individual samples using Trizol reagent (Invitrogen, Carlsbad, CA, USA) according to the manufacturer’s instructions. Then cDNA was synthesised from 100 ng RNA using iScript^TM^ cDNA Synthesis Kit (Bio-Rad). Quantitative PCR was performed on the ABI 7900HT (Applied Biosystems) using the iTaq^TM^ Universal SYBR^®^ Green Supermix (Bio-Rad) in a 96-well plate (20-μl reactions). All primers are listed in Supplementary Table 4. Beta-actin (β-actin) was used as the reference gene^50^. The relative quantification was analysed using the 2^–ΔΔCT^ method^51^.

### Statistical analysis

For learning speed assessment, a learning curve was obtained by fitting a first-order exponential decay function to the number of errors in each ten landings for each bee (see details in our published work^9,52^). The decay constant (*t*) calculated from the function is the measure of learning speed: high *t* values correspond to slow learning, whereas lower *t* values represented faster learning. For memory retention assessment, the proportion of landings on rewarding flowers in the retention test for each bee was calculated.

Spearman’s rank correlation coefficient was applied to examine the relationships between the cognitive performance and the abundance of specific genus or phylotype (RPKM values from metagenomic analysis), the absolute abundance of total gut bacteria (16 S rRNA gene copies), and the abundance of functional terms (KEGG pathways and KO terms, RPKM values). The analysis was confined to functional terms that were shared by >20% of the samples. Bonferroni corrections were performed to give an indication of false-positive significances given multiple tests, and are presented in Supplementary Data files. Although this conservative correction reduced the significance of all *p* values, the Spearman’s Rho values (*r*) signify the correlations between memory and bacteria/functional terms. Partial correlation analysis was conducted to validate the relationships between memory retention and the five genera. Generalised linear mixed models (GLMM) with binomial distribution and link function ‘logit’ were applied to examine the effects of different treatments (*L. apis*, *S. alvi*, *G. apicola* and LPA) on memory retention. GLMMs with gamma distribution and link function ‘reciprocal’ were used to examine the effects of different treatments on learning speed. Mann-Whitney *U* test and Student’s *t* test were used to compare the bacteria abundance and gene expression of neural receptors between experimental and control groups. All behaviour data are shown in Supplementary Data 12. The colony and bee information for each of the experiments is provided in Supplementary Table 5. Statistical tests (all two-sided) were performed with MATLAB (R2017a) and R (Version 3.4.3). The significance level used was 5% in all analyses.

### Reporting summary

Further information on research design is available in the Nature Research Reporting Summary linked to this article.

## Supplementary information

Supplementary information

Description of Additional Supplementary Files

Supplementary Data 1

Supplementary Data 2

Supplementary Data 3

Supplementary Data 4

Supplementary Data 5

Supplementary Data 6

Supplementary Data 7

Supplementary Data 8

Supplementary Data 9

Supplementary Data 10

Supplementary Data 11

Supplementary Data 12

Supplementary Movie 1

Supplementary Movie 2

Supplementary Movie 3

Reporting Summary

## Data Availability

The metagenomic sequencing data have been deposited in the NCBI Sequence Read Achieve database under BioProject accession number PRJNA699677. The raw metabolomic data have been deposited in the MetaboLights with the identifier MTBLS3637. All data supporting the findings of this study are available in the manuscript or supplementary information. Source data are provided with this paper.

## Source data

Source Data
