# Supplementary material for: Gut microbiome drives individual memory variation in bumblebees
Source: Nat Commun. 2021 Nov 25;12:6588. doi: 10.1038/s41467-021-26833-4 (PMC8616916; doi:10.1038/s41467-021-26833-4)
Supplement: Supplementary file 1 — Supplementary information [file 41467_2021_26833_MOESM1_ESM.pdf]

Gut microbes as a driver for individual memory variation in bumblebees

Li Li[1], Cwyn Solvi[2], Feng Zhang[3], Zhaoyang Qi[1], Lars Chittka[2], Wei Zhao[1]*

*Correspondence: zhaow@jiangnan.edu.cn

Supplementary information

Supplementary Methods

Supplementary Figures 1-10

Supplementary Tables 1-5

Supplementary References

Supplementary Methods

Our gene mapping method could not with certainty identify individual bacterial species because not all the 31 housekeeping genes were detected and with > 95% sequence identity (Methods, Supplementary Data 10). Therefore, three other approaches (mOTU, MetaPhlAn3 and Kraken2) were performed.

For mOTU, the high-quality sequencing reads obtained from the gene-mapping approach were aligned to the mOTU database with default parameters by the mOTU pipeline (Version 3.0.1)[1], identifying species (mOTUs) and their relative abundance. The mOTU database contains phylogenetic marker gene sequences extracted from reference genomes, metagenomic samples and metagenome assembled genomes. Like the gene-mapping method, mOTU also identified the four dominant species known to exist in bee guts (Supplementary Data 11 and Supplementary Fig. 9). They were *S. alvi*, *G. apicola*, *B. bohemicum* and *Lactobacillus* Firm-5 species (*L. apis* identified by the gene-mapping method and *L. bombicola* identified by mOTU). In our study, *Lactobacillus* strains were isolated from the hindguts of bumblebees (*Bombus terrestris*) using MRS agar. Four of the 20 isolates were *L. apis* strains, 12 of them were unclassified *Lactobacillus*, and none of them were *L. bombicola*. This indicates that *L. apis* may be the most abundant classified *Lactobacillus* species in our bumblebees. Further, for this reason, we cannot be confident that the mOTU method identified individual bacterial species accurately.

The high-quality sequencing reads were also used for species-level taxonomic annotation with another two established tools, MetaPhlAn3[2] and Kraken2[3]. MetaPhlAn3 (Version 3.0.2) was performed with default parameters, which is based on unique clade-specific marker genes. Kraken2 (Version 2.1.1-beta) was conducted for taxonomic annotation with default parameters, which is based on exact k-mer assignments. The Minikraken database (MiniKraken2_v2_8GB) was downloaded

(ftp://ftp.ccb.jhu.edu/pub/data/kraken2_dbs/old/minikraken2_v2_8GB_201904.tgz) and used. The relative abundances of taxa were subsequently calculated by Bracken (Version 3.29)[4]. Our quantitative real-time PCR results detected the known dominant bacterial species in bumblebee guts, including *S. alvi*, *G. apicola* and *B. bohemicum*, which were shown to be highly abundant (Supplementary Fig. 10). However, MetaPhlAn3 did not identify *S. alvi* and *G. apicola*, and Kraken2 did not identify *B. bohemicum* (Supplementary Data 11 and Supplementary Fig. 9). Therefore, we again cannot be confident that these two approaches accurately identified bacterial species.

Lactobacillus Firm-5 is one dominant phylotype in honeybee and bumblebee guts[5,6]. Phylogenetic analyses of Ellegaard et al.[6] indicate that Firm-5 strains isolated from honeybees and bumblebees belong to separate sublineages of the Firm-5 phylotype. Their results suggest that *L. apis* is specific to honeybees and *L. bombicola* is specific to bumblebees. However, we isolated 4 *L. apis* strains (no *L. bombicola* strains) out of 20 isolates from bumblebee guts, demonstrating the existence of *L. apis* in bumblebee guts and indicating its possible high abundance among the classified *Lactobacillus*. The differences found between their study and ours may be because the bumblebees used in each study were different species/subspecies from different regions and environment (wild bumblebees in western Switzerland versus indoor-kept commercial bumblebees from Koppert, China). Different environments and diets will lead to distinct gut microbiota composition, including different bacterial species and their abundance.

a

Human colour	+	+	-	+	-	+	-	+	-	+	-
RGB values	242,242,242	255,1,1	255,152,1	243,255,1	152,255,1	1,255,61	1,255,213	1,182,255	61,1,255	182,1,255	255,1,213
Hexagon loci (x,y)	-0.0819, 0.2193	-0.1916, 0.0392	0.0523, 0.0414	0.3316, -0.0407	0.4018, 0.0101	0.3790, 0.0238	0.3290, 0.0297	0.3025, 0.2003	0.2486, 0.2533	0.0026, 0.3127	-0.0685, 0.2628

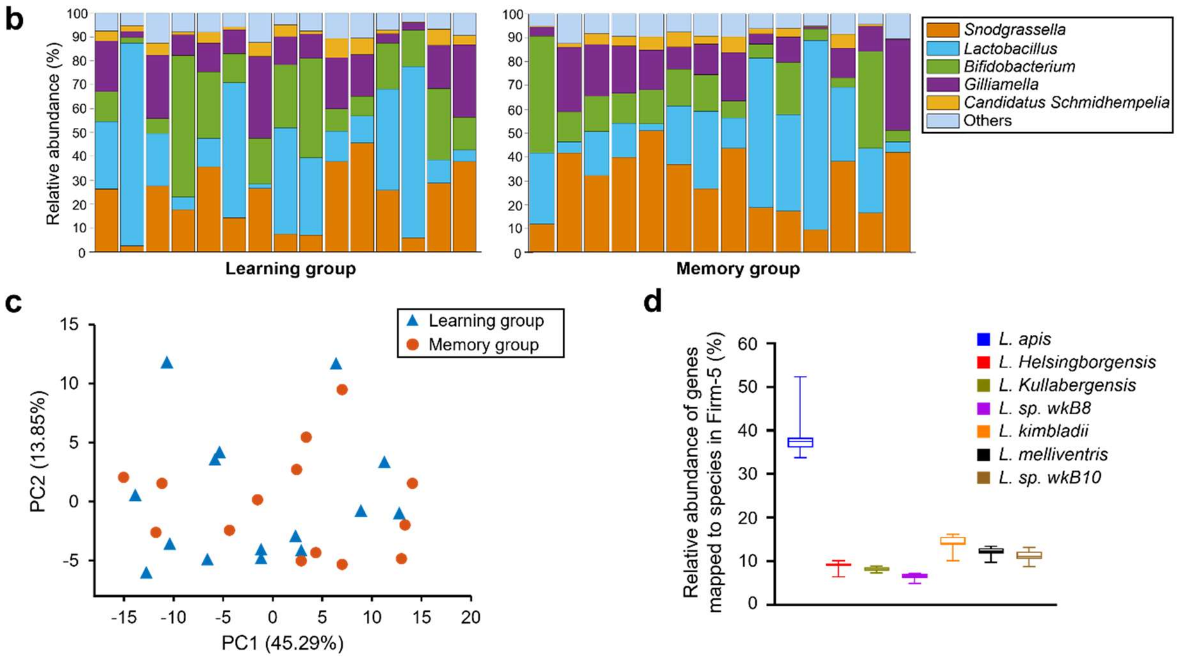

Supplementary Fig. 1 Colours used for the 10-Colour Learning paradigm and the structural characteristics of the bumblebee gut microbiota. a, Colours of artificial flowers in the learning task are shown as they are perceived by humans, with human RGB values underneath and coordinates of colour loci in the bee-subjective hexagon colour space in the 3rd row. +/- symbols indicate rewarding (+) and unrewarding (-) chips during training. **b,** The gut microbiota composition (genus level) of individual bees in the Learning group (n = 15) and the Memory group (n = 14). Each column represents an individual bee. Inter-individual variation for bee gut microbiota composition is shown at the genus level. **c,** Principal component analysis was performed based on the abundance of gut bacterial genera (RPKM values), showing that the gut microbiota composition for bees in the Learning group was similar to that for bees in the Memory group (PERMANOVA: $R^2 = 0.0364$, $p = 0.378$). **d,** The relative abundance of genes that were mapped to each Firm-5 species in the Firm-5 community. The percentage was calculated as the abundance of genes mapped to a Firm-5 species divided by the total abundance of genes mapped to the Firm-5 community (RPKM values), to indicate which Firm-5 species may be the most abundant. In the boxplot, the boxes show medians, lower and upper quartiles, and the whiskers extend to the highest and lowest values (n = 14 bees). Source data are provided as a Source Data file.

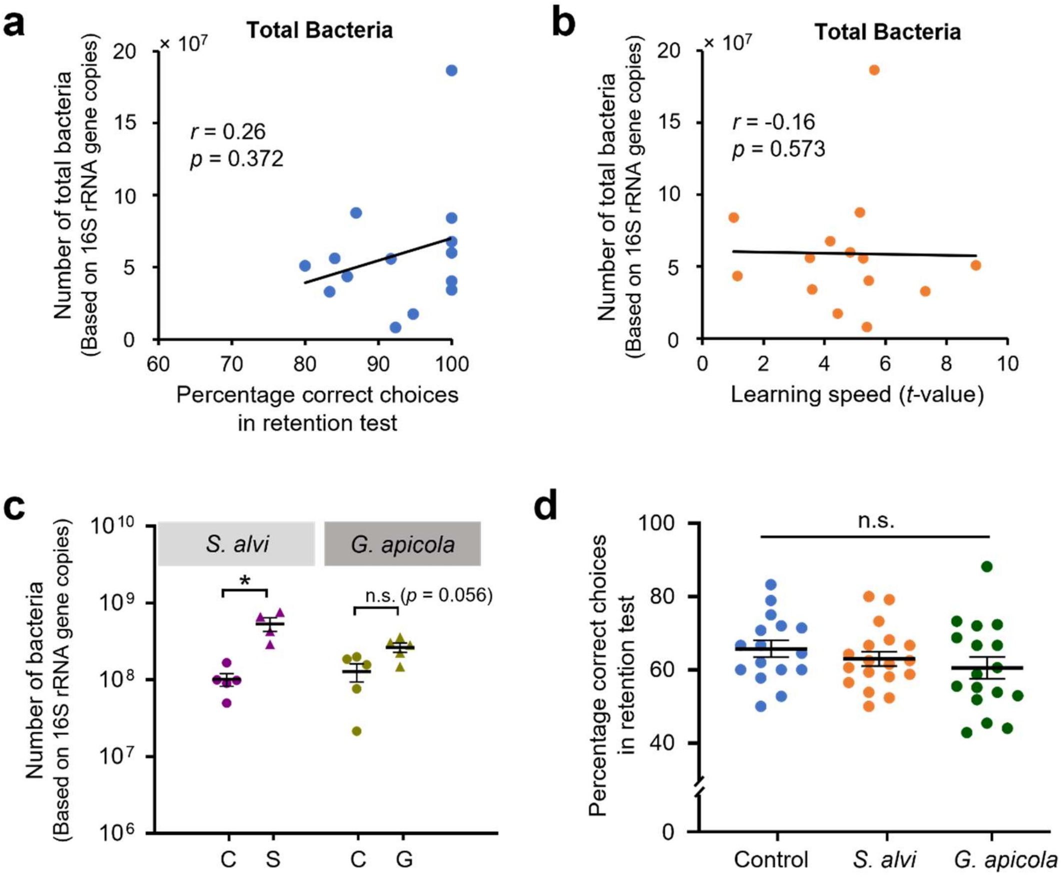

Supplementary Fig. 2 Learning and memory are not affected by total gut bacteria, or by increases in *S. alvi* or *G. apicola*. **a** and **b**, Long-term memory and learning speed did not correlate with the absolute abundance of total bacteria in the bee hindgut. The *t* value was the indicator for learning speed (Methods). High *t* values indicate slow learning whereas low *t* values indicate fast learning. Spearman correlation analysis (two-sided) was conducted, and the coefficient *r* value and significance *p* value are shown in each panel, $n = 14$ bees. **c**, *S. alvi* supplemented diet increased its abundance in the bee hindgut significantly (two-sided Mann-Whitney *U* test: $U = 0$, $n = 5$ bees for the Control group, $n = 4$ bees for the *S. alvi* supplemented group, $p = 0.016$); whereas *G. apicola* supplemented diet had a positive but non-significant effect ($U = 3$, $n = 5$ bees for both groups, $p = 0.056$). C: Control group fed with sugar without bacteria; S: *S. alvi* supplemented diet; G: *G. apicola* supplemented diet. **d**, Supplementation of *S. alvi* ($n = 18$) or *G. apicola* ($n = 17$) did not affect bee long-term memory compared to Control (sugar without added bacteria; $n = 16$) (GLMM, Supplementary Table 2). In **c** and **d**, data are presented as mean ± SEM, and asterisks indicate significant differences ($*p < 0.05$). n.s.: not significant. Source data are provided as a Source Data file.

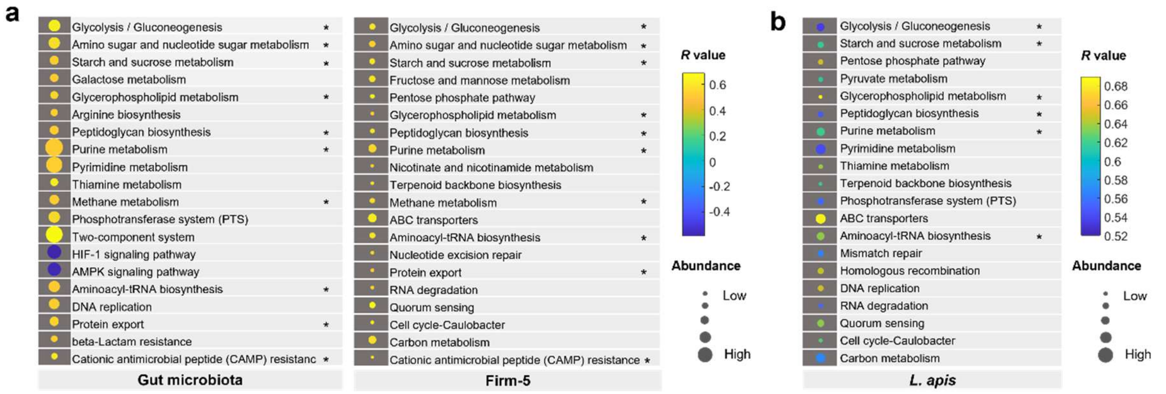

Supplementary Fig. 3 Metagenomic analysis identified KEGG pathways correlated with long-term memory. **a**, Long-term memory correlated KEGG pathways found based on their abundance in the whole gut microbiota or in the Firm-5. **b**, Long-term memory correlated KEGG pathways found based on the genes mapped to *L. apis*. Spearman correlation analyses were conducted separately for whole gut microbiota, Firm-5 and *L. apis*, $n = 14$ bees. The 20 most abundant memory correlated KEGG pathways are shown here. The colours of the circles indicate the coefficient r values and the sizes of the circles indicate the average abundance of the KEGG pathways. Several long-term memory correlated pathways were shared by the whole gut microbiota and Firm-5 labelled by asterisk in **a**, some of which were also found memory correlated in *L. apis*, suggesting their vital roles in long-term memory regulation. Source data are provided as a Source Data file.

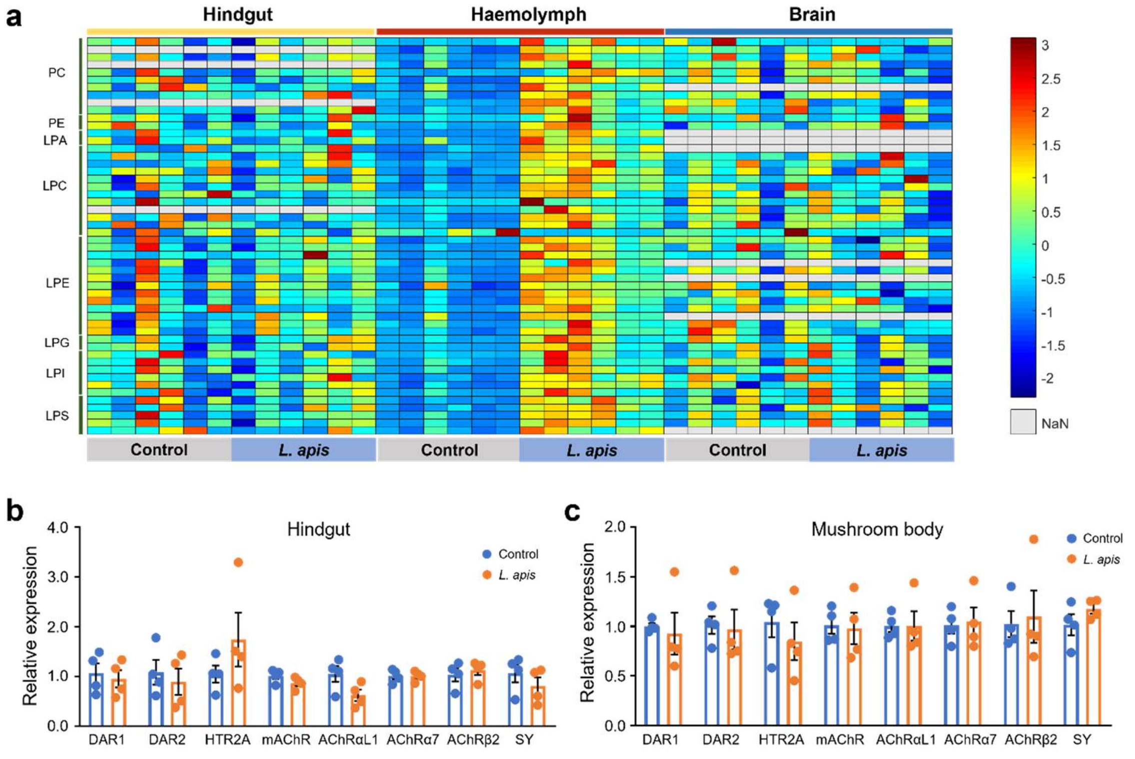

Supplementary Fig. 4 *L. apis* supplementation affects glycerophospholipid metabolism and the expression of neural receptors. a, A heatmap shows the abundance of the glycerophospholipids in the bee hindgut, haemolymph and brain. Colours indicate the normalized abundance of each metabolite. *L. apis* supplementation increased glycerophospholipids abundance in the haemolymph significantly, but did not affect their abundance in the hindgut and brain (Supplementary Data 7). NaN (grey colour): the glycerophospholipids were not found in one of the tissues. **b** and **c**, The effects of *L. apis* administration on the gene expression of neural receptors in the host hindgut and brain mushroom body (n = 4 bees for both groups). Most of the checked neural receptors were not affected by *L. apis* supplementation (two-sided Student's t-test: $p > 0.05$). Data are presented as mean ± SEM. DAR1, dopamine receptor 1; DAR2, dopamine receptor 2; HTR2A, 5-hydroxytryptamine receptor 2A; mAChR, muscarinic acetylcholine receptor DM1; AChRαL1, acetylcholine receptor subunit alpha-L1; AChRα7, neuronal acetylcholine receptor subunit alpha-7; AChRβ2, acetylcholine receptor subunit beta-like 2; SY, synapsin. Source data are provided as a Source Data file.

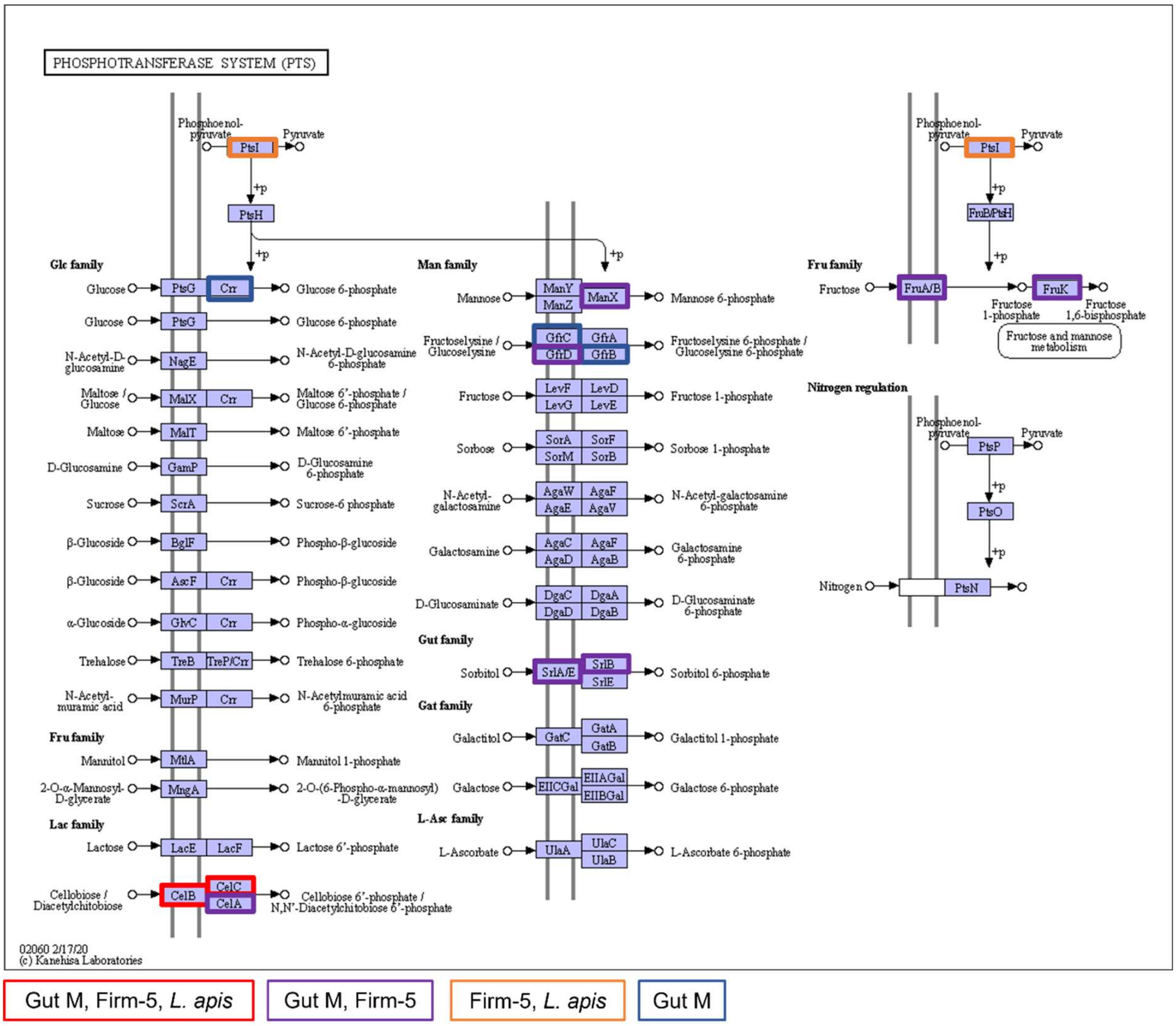

Supplementary Fig. 5 Memory-correlated genes found in the pathway Phosphotransferase system (PTS). The gene abundance was calculated based on genes mapped to the entire gut microbiota (Gut M), Firm-5 and *L. apis*, respectively. The correlation analyses were conducted at the three different levels and different colours label the memory-correlated genes found in which level analysis.

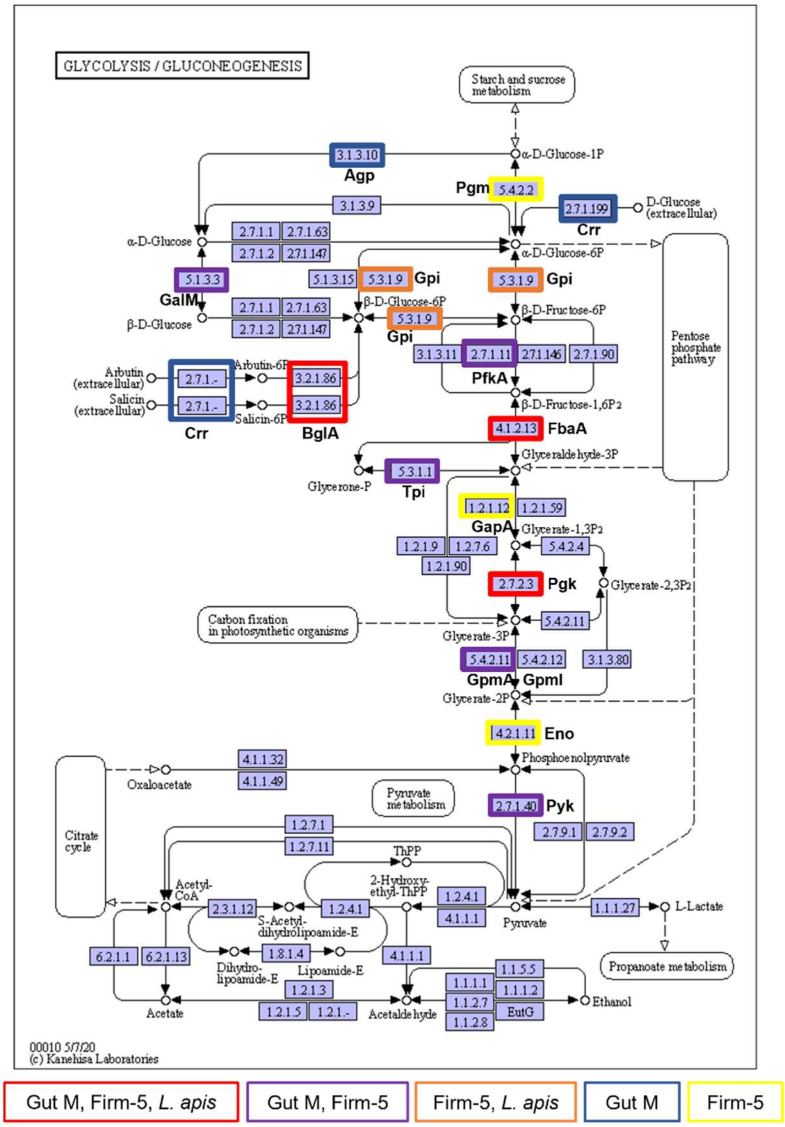

Supplementary Fig. 6 Memory-correlated genes found in the pathway Glycolysis/Gluconeogenesis. The gene abundance was calculated based on genes mapped to the entire gut microbiota (Gut M), Firm-5 and *L. apis*, respectively. The correlation analyses were conducted at the three different levels and different colours label the memory-correlated genes found in which level analysis.

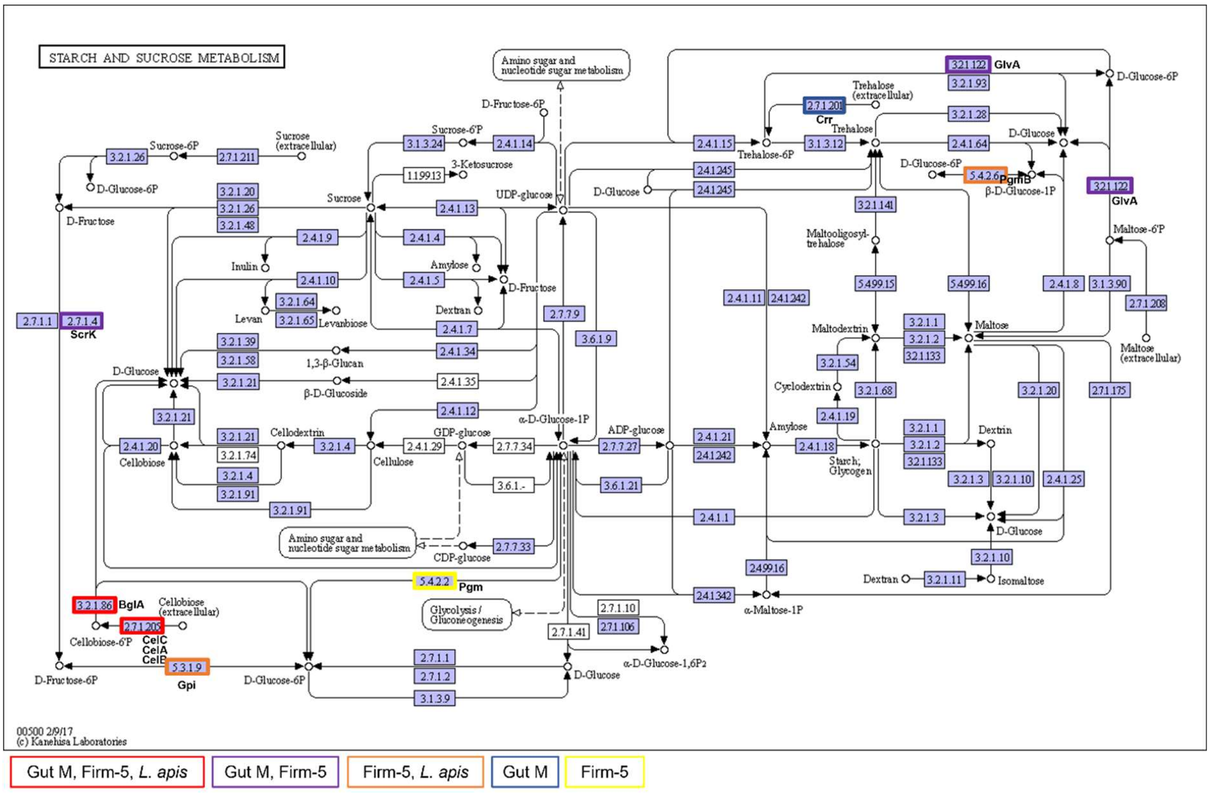

Supplementary Fig. 7 Memory-correlated genes found in the pathway Starch and sucrose metabolism. The gene abundance was calculated based on genes mapped to the entire gut microbiota (Gut M), Firm-5 and *L. apis*, respectively. The correlation analyses were conducted at the three different levels and different colours label the memory-correlated genes found in which level analysis.

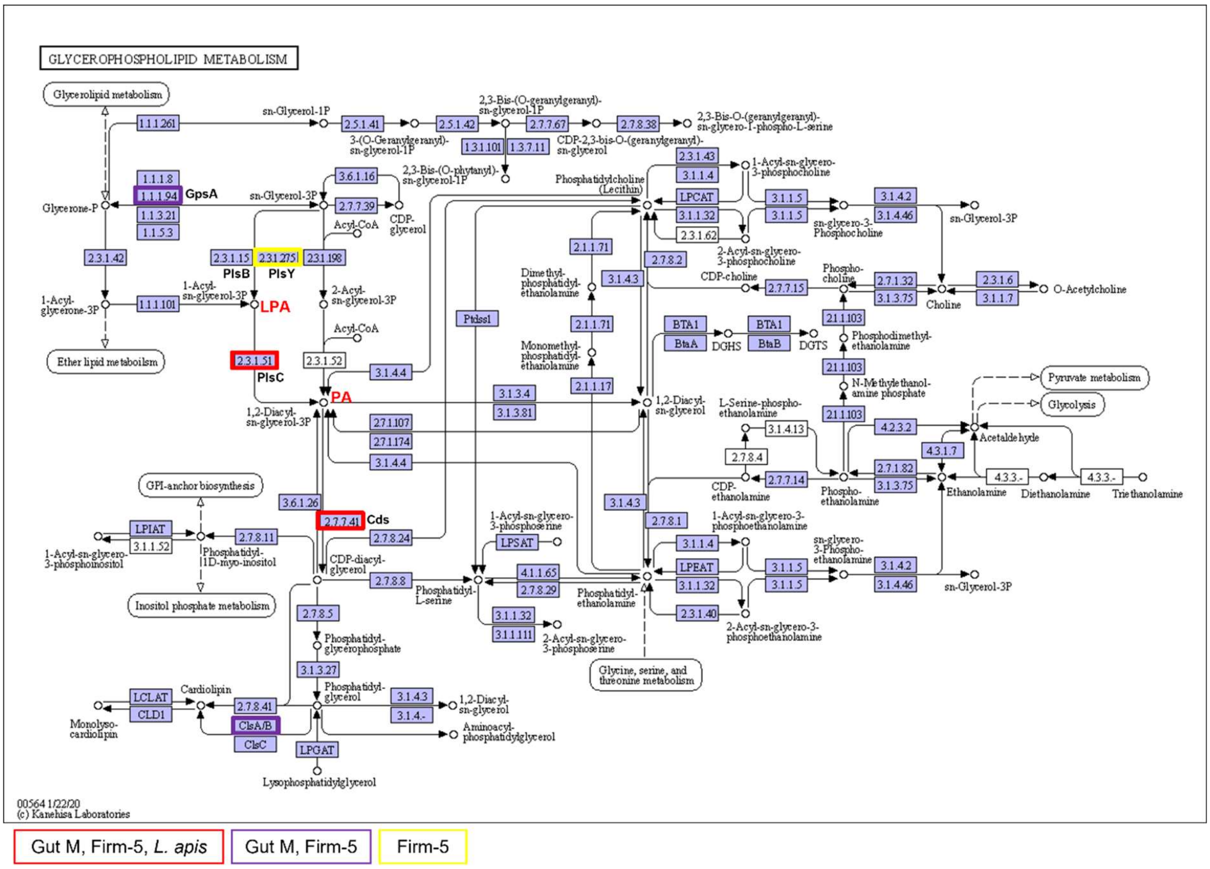

Supplementary Fig. 8 Memory-correlated genes found in the pathway Glycerophospholipid metabolism. Gene abundance was calculated based on genes mapped to the entire gut microbiota (Gut M), Firm-5 and *L. apis*, respectively. The correlation analyses were conducted at the three different levels and different colours label the memory-correlated genes found in which level analysis.

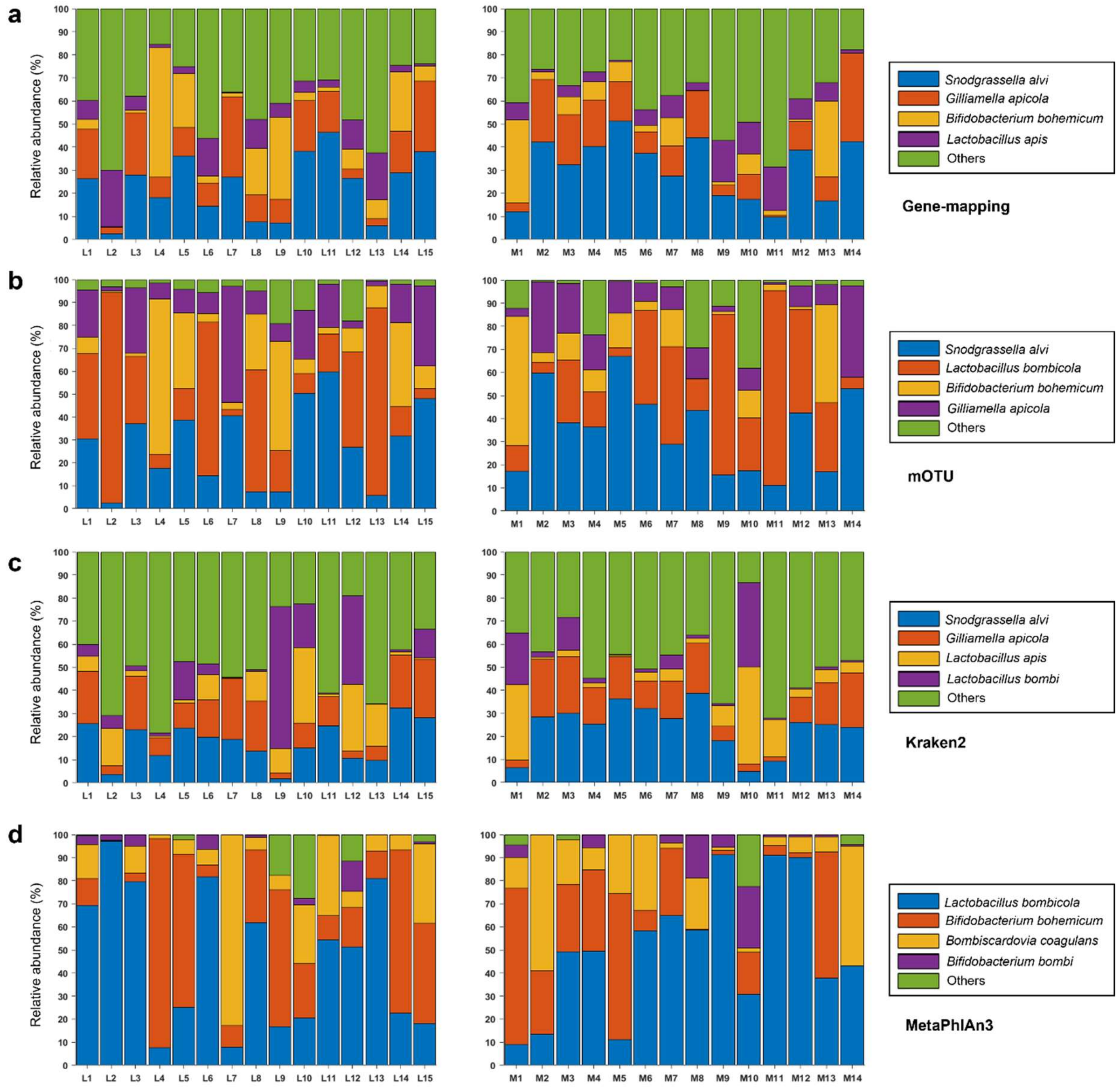

Supplementary Fig. 9 The gut microbiome composition of individual bees determined with different methods. a-d, The bacterial species were identified with the gene-mapping method, mOTU, Kraken2 and MetaPhlAn3, respectively. Each column represents an individual bee (Learning group, *n* = 15; Memory group, *n* = 14). Only the four most abundant species found for each method are shown. Source data are provided as a Source Data file.

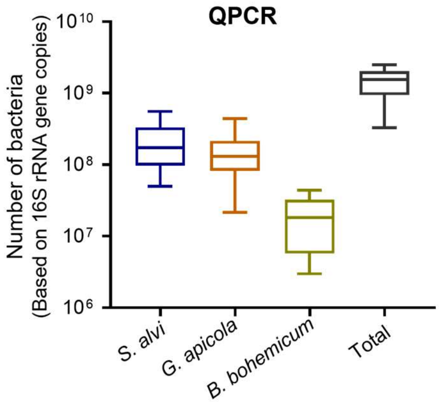

Supplementary Fig. 10 The absolute abundance of gut bacteria in bumblebee hindguts determined by quantitative real-time PCR. *S. alvi*, *G. apicola* and *B. bohemicum*, three known dominant bacterial species in the bumblebee gut, were detected and shown to be highly abundant (compared with the total abundance of gut bacteria). *N* = 10. In the boxplot, the boxes show medians, lower and upper quartiles, and the whiskers extend to the highest and lowest values. Source data are provided as a Source Data file.

Supplementary Table 1 Summary of generalized linear mixed models examining *L. apis* treatment factors in relation to memory retention and learning speed.

Dependent variable	Fixed factors	df	Estimate	SE	*t*	*p*
Memory retention	Intercept	64	0.1951	0.3278	0.60	0.554
	**Treatment (*L. apis*)**	64	0.9628	0.2170	4.44	**$3.656e^{-5}$**

The dependent variable was the percentage correct choices during the memory retention test. The treatment was fixed factor. Age, colony (n = 2) and bee ID were included as random factors. The reference treatment was the bees feeding with sugar solution without *L. apis*. The significant terms are highlighted in bold.

Dependent variable	Fixed factors	df	Estimate	SE	*t*	*p*
Learning speed	Intercept	64	0.4326	0.1277	3.39	0.001
	Treatment (*L. apis*)	64	-0.0324	0.0689	-0.47	0.640

The dependent variable was the t-value calculated for learning speed during training. The treatment was fixed factors. Age, colony (n = 2), bee ID and number of landings were included as random factors. The reference treatment was the bees feeding with sugar solution without *L. apis*.

Supplementary Table 2 Summary of generalized linear mixed models examining *S. alvi* and *G. apicola* treatment factors in relation to memory retention and learning speed.

Dependent variable	Fixed factors	df	Estimate	SE	*t*	*p*
Memory retention	Intercept	48	0.5959	0.1014	5.88	$3.835e^{-7}$
	Treatment (*S. alvi*)	48	-0.0894	0.1376	-0.65	0.519
	Treatment (*G. apicola*)	48	-0.1815	0.1461	-1.24	0.220

The dependent variable was the percentage correct choices during the memory retention test. The treatments were fixed factors. Age and bee ID were included as random factors. Colony was not included as the random factor because all bees were from the same colony. The reference treatment was the bees feeding with sugar solution without bacteria.

Dependent variable	Fixed factors	df	Estimate	SE	*t*	*p*
Learning speed	Intercept	48	0.1957	0.0258	7.57	$9.779e^{-10}$
	Treatment (*S. alvi*)	48	0.0520	0.0372	1.40	0.169
	Treatment (*G. apicola*)	48	0.0585	0.0410	1.43	0.160

The dependent variable was the t-value calculated for learning speed during training. The treatments were fixed factors. Age, bee ID and number of landings were included as random factors. Colony was not included as the random factor because all bees were from the same colony. The reference treatment was the bees feeding with sugar solution without bacteria.

Supplementary Table 3 Summary of generalized linear mixed models examining the glycerophospholipid LPA (14:0) treatment factors in relation to memory retention.

Dependent variable	Fixed factors	df	Estimate	SE	*t*	*p*
Memory retention	Intercept	24	0.0510	0.3352	0.15	0.880
	Treatment (LPA 300 μM)	24	0.5106	0.2187	2.33	**0.028**

The dependent variable was the percentage correct choices during the memory retention test. The treatment was fixed factor. Age and bee ID were included as random factors. Colony was not included as the random factor because all bees were from the same colony. The reference treatment was the bees feeding with sugar solution without LPA.

Supplementary Table 4 Primers used in QPCR.

Bacteria	Target	Forward primer	Reverse primer
S. alvi	16S rRNA	CTTAGAGATAGGAGAGTGCCTT	AACTTAATGATGGCAACTAATGACAA
G. apicola	16S rRNA	CTTTGTTGCCATCGGTTAGGCC	CCGCTTGCTCTCGCGAGG
B. bohemicum	16S rRNA	AGATGTCGTTTCCCTTCGGG	TGTGAGTTCCCGGCATAACC
Firm 4	16S rRNA	AGTCGAGCGCGGGAAGTCA	AGCCGTCTTTCAACCAGCACT
Firm 5	16S rRNA	GGAATACTTCGGTAGGAA	CTTATTTGGTATTAGCACC
Universal bacteria	16S rRNA	AGAGTTTGATCCTGGCTCAG	CTGCTGCCTCCCGTAGGAGT
**Gene**	**NCBI accession number**	**Forward primer**	**Reverse primer**
Actin	XM_003396941.3	TGACGCAGATTATGTTTGAA	AGCGTATAGCGAAAGTACAGC
mGlu2	XM_012315392.2	GGCATTCAAAGATTTGCCGC	GCTGTGTTTGCTCGCATTGA
NR2B	XM_020863765.1	CATCCGCTACCGCCATACTT	CGTCGTCCGGAATCCTGTAG
OARO	XM_012316931.2	AAACGGGGGATGAGATGTCG	TCGGAATCTTTTCACCTGAGCT
OARβ1	XM_012310827.2	ACCTCGAGACTCCGAAGATGT	ACGTTGCACGGTTGCATTTC
OARβ2	XM_012318605.2	TCACAGCGTTGAATGTAACCAC	TCAGAATCGTGTACGGTGGC
DAR1	XM_020867093.1	TGGGGATTTATTGCAGGCTCT	GCTTCGTTTTTGTTTGCGGC
DAR2	XM_012316882.2	TGTCCCTCTCCCTCTCTGAC	GTTTGTCCCCCGGAGATACG
HTR2A	XM_020864397.1	GCCCTGCGATTACTCCAACT	GGCGTGCATTCCAAAATCTGA
mAChR	XM_003401716.2	ATTCTAGCGATGAAGCGTTGG	GTCGCATTCATGTCGTCAGC
AChRαL1	XM_003397511.3	GGACTTCGTCTGTCCCAACT	GGTCTTGCCATTCGTGTTCG
AChRα7	XM_012312542.2	ATACAACGTGCTGGAGCGTC	GGTTCTTCTCGTCAACGTCG
AChRβ2	XM_012308355.2	AAGTGTCTTGGAAACCGCCT	GATCCACCTGAGCGCCATT
SY	XM_020866077.1; XM_003402975.3	CGGTGTCAGGTGCAACTAGA	ATTCCGCCTGTTCTACACGG

Supplementary Table 5 The summary of colony and bee information for each of our experiments.

Experiment ID	Aim of the experiment	Colony	Groups	Number of bees in each group	Other descriptions
1	To examine correlations between learning/memory abilities and the gut microbiota (Metagenomic sequencing and QPCR)	Colony A	Learning group	15	Total genomic DNA was extracted from each bee hindgut, which was used for metagenomic sequencing and quantifying gut total bacteria by QPCR.
			Memory group	14	
2	To examine the effect of *L. apis* supplementation on long-term memory	Colony B	Control group	12	Bacteria abundance was determined by QPCR.
			L. apis group	12	
		Colony C	Control group	20	Brains and hindguts were used for neural receptors' determination.
			L. apis group	22	
3	To examine the effect of *S. alvi* and *G. apicola* supplementation on long-term memory	Colony D	Control group	16	Bacteria abundance was determined by QPCR.
			S. alvi group	18	
			G. apicola group	17	
4	To examine the effect of *L. apis* supplementation on host metabolites' changes by untargeted metabolomic analysis	Colony E	Control group	37	The hindguts, haemolymph or brains from 11 to 14 bees were pooled in each sample and there were three biological replicates from Colony E
			L. apis group	35	The hindguts, haemolymph or brains from 11 to 14 bees were pooled in each sample and there were three biological replicates from Colony E
		Colony F	Control group	35	The hindguts, haemolymph or brains from 11 to 14 bees were pooled in each sample and there were three biological replicates from Colony F
			L. apis group	40	The hindguts, haemolymph or brains from 11 to 14 bees were pooled in each sample and there were three biological replicates from Colony F
5	To examine the effect of LPA (300 μM) supplementation on long-term memory	Colony G	Control group	13	
			LPA (300 μM) group	14	

Supplementary References

1. Milanese, A. *et al.* Microbial abundance, activity and population genomic profiling with mOTUs2. *Nat. Commun.* **10**, 1014 (2019).
2. Beghini, F. *et al.* Integrating taxonomic, functional, and strain-level profiling of diverse microbial communities with bioBakery 3. *Elife* **10**, 1–42 (2021).
3. Wood, D. E., Lu, J. & Langmead, B. Improved metagenomic analysis with Kraken 2. *Genome Biol.* **20**, 257 (2019).
4. Lu, J., Breitwieser, F. P., Thielen, P. & Salzberg, S. L. Bracken: estimating species abundance in metagenomics data. *PeerJ Comput. Sci.* **3**, e104 (2017).
5. Kwong, W. K. & Moran, N. A. Gut microbial communities of social bees. *Nat. Rev. Microbiol.* **14**, 374–384 (2016).
6. Ellegaard, K. M. *et al.* Genomic changes underlying host specialization in the bee gut symbiont Lactobacillus Firm5. *Mol. Ecol.* **28**, 2224–2237 (2019).
