# Supplementary material for: Gut microbiome drives individual memory variation in bumblebees
Source: Nat Commun. 2021 Nov 25;12:6588. doi: 10.1038/s41467-021-26833-4 (PMC8616916; doi:10.1038/s41467-021-26833-4)
Supplement: Supplementary file 2 — Description of Additional Supplementary Files [file 41467_2021_26833_MOESM2_ESM.docx]

**Gut microbes as a driver for individual memory variation in bumblebees**

Li Li^1^, Cwyn Solvi^2^, Feng Zhang^3^, Zhaoyang Qi^1^, Lars Chittka^2^, Wei Zhao^1^*

*Correspondence: zhaow@jiangnan.edu.cn

**Supplementary information**

Supplementary Methods

Supplementary Figures 1-10

Supplementary Tables 1-5

Supplementary References

**Supplementary files**

Supplementary Data 1-12

Supplementary Movies 1-3

**Supplementary files:**

**Supplementary Data 1.** The summary of sequencing data obtained from metagenomic sequencing of individual hindguts.

**Supplementary Data 2.** The abundance of gut bacteria identified in individual bee guts from the Learning group (*n* = 15) and the Memory group (*n* = 14) based on bacterial RPKM from metagenomic sequencing. The five most dominant genera and five main phylotypes are shown.

**Supplementary Data 3.** The correlations between learning/memory performance and the abundance of gut bacteria (RPKM values). Spearman’s rank correlation analyses with Bonferroni correction were conducted.

**Supplementary Data 4.** The abundance of KEGG pathways identified for *Lactobacillus* Firm-5, *S. alvi* and *G. apicola*. The top 50 most abundant KEGG pathways are shown.

**Supplementary Data 5.** The correlations between long-term memory retentions and KEGG pathways. Spearman’s rank correlation analyses with Bonferroni correction were conducted.

**Supplementary Data 6.** The significantly altered metabolites in the bumblebee hindgut, haemolymph and brain after *L. apis* supplementation.

**Supplementary Data 7.** The abundance of glycerophospholipids and lysoglycerophospholipids in the hindgut, haemolymph and brain of bumblebees with or without *L. apis* supplementation.

**Supplementary Data 8.** The long-term memory correlated genes in KEGG pathways of Phosphotransferase system (PTS), Glycolysis/Gluconeogenesis, Starch and sucrose metabolism and Glycerophospholipid metabolism.

**Supplementary Data 9.** The primary data for bacteria taxonomic annotation.

**Supplementary Data 10.** The species identification by analysing the housekeeping genes of each of the four most abundant species.

**Supplementary Data 11.** The relative abundance of gut bacterial species identified in individual bee guts with different approaches (gene-matching method, mOTU, Kraken2 and MetaPhlAn3).

**Supplementary Data 12.** Behaviour data used for statistical analyses.

**Supplementary Movie 1.** Training in 10 Colour Learning. Bees were trained to discriminate five different rewarding (sucrose solution) colours from five different punishing (bitter quinine solution) colours. On the last trip, bees learned the task, landing more times to the rewarding colours. The rewarding (+) and unrewarding (-) colours are labelled in the video.

**Supplementary Movie 2.** A bee showing good performance in the memory retention test. In the test, all flowers contained water and bee landings to flowers within three minutes were recorded to evaluate memory retention. +/- symbols in the video indicate rewarding (+) and unrewarding (-) colours during training.

**Supplementary Movie 3.** A bee showing poor performance in the memory retention test. In the test, all flowers contained water and bee landings to flowers within three minutes were recorded to evaluate memory retention. +/- symbols in the video indicate rewarding (+) and unrewarding (-) colours during training.
