# Supplementary material for: Gut microbiome drives individual memory variation in bumblebees
Source: Nat Commun. 2021 Nov 25;12:6588. doi: 10.1038/s41467-021-26833-4 (PMC8616916; doi:10.1038/s41467-021-26833-4)
Supplement: Supplementary file 18 — Reporting Summary [file 41467_2021_26833_MOESM18_ESM.pdf]

nature portfolio

Corresponding author(s): Wei Zhao

Last updated by author(s): Oct 20, 2021

Reporting Summary

Nature Portfolio wishes to improve the reproducibility of the work that we publish. This form provides structure for consistency and transparency in reporting. For further information on Nature Portfolio policies, see our Editorial Policies and the Editorial Policy Checklist.

Statistics

For all statistical analyses, confirm that the following items are present in the figure legend, table legend, main text, or Methods section.

n/a	Confirmed	
☐	☒	The exact sample size (*n*) for each experimental group/condition, given as a discrete number and unit of measurement
☐	☒	A statement on whether measurements were taken from distinct samples or whether the same sample was measured repeatedly
☐	☒	The statistical test(s) used AND whether they are one- or two-sided<br>*Only common tests should be described solely by name; describe more complex techniques in the Methods section.*
☐	☒	A description of all covariates tested
☐	☒	A description of any assumptions or corrections, such as tests of normality and adjustment for multiple comparisons
☐	☒	A full description of the statistical parameters including central tendency (e.g. means) or other basic estimates (e.g. regression coefficient) AND variation (e.g. standard deviation) or associated estimates of uncertainty (e.g. confidence intervals)
☐	☒	For null hypothesis testing, the test statistic (e.g. *F*, *t*, *r*) with confidence intervals, effect sizes, degrees of freedom and *P* value noted<br>*Give P values as exact values whenever suitable.*
☒	☐	For Bayesian analysis, information on the choice of priors and Markov chain Monte Carlo settings
☒	☐	For hierarchical and complex designs, identification of the appropriate level for tests and full reporting of outcomes
☐	☒	Estimates of effect sizes (e.g. Cohen's *d*, Pearson's *r*), indicating how they were calculated

Our web collection on statistics for biologists contains articles on many of the points above.

Software and code

Policy information about availability of computer code

Data collection	For metagenomic data collection, NovaSeq™ 6000 Sequencing System Control Software (Version 1.7.0) was used. For metabolomic data collection, Xcalibur 4.1 (Thermo Fisher) and Q Exactive HF-X Tune 2.9 (Thermo Fisher) were used. For quantitative real-time PCR data collection, ABI 7900HT Sequence Detection Systems (Version 2.4) and QuantStudio™ Design & Analysis Software (Version 1.3.1) were used.
Data analysis	Sickle (Version 1.33), BWA (Version 0.7.17-r1188), MEGAHIT (Version 1.1.2), MetaGene, CD-HIT (Version 4.6.1), SOAPaligner (Version 2.22), DIAMOND (Version 0.8.35), UBCG (Version 3.0), mOTU (Version 3.0.1), MetaPhlAn3 (Version 3.0.2) and Kraken2 (Version 2.1.1-beta) were used for metagenomic analysis. Compound Discoverer 3.1 (Thermo Fisher) was used for metabolomic analysis. Statistical analyses were performed with MATLAB (R2017a) and R (Version 3.4.3).

For manuscripts utilizing custom algorithms or software that are central to the research but not yet described in published literature, software must be made available to editors and reviewers. We strongly encourage code deposition in a community repository (e.g. GitHub). See the Nature Portfolio guidelines for submitting code & software for further information.

Data

Policy information about availability of data

All manuscripts must include a data availability statement. This statement should provide the following information, where applicable:

- Accession codes, unique identifiers, or web links for publicly available datasets
- A description of any restrictions on data availability
- For clinical datasets or third party data, please ensure that the statement adheres to our policy

The metagenomic sequencing data have been deposited in the NCBI Sequence Read Achieve database under BioProject accession number PRJNA699677 (https://www.ncbi.nlm.nih.gov/sra/PRJNA699677). The raw metabolomic data have been deposited in the MetaboLights with the identifier MTBLS3637 (www.ebi.ac.uk/

metabolights/MTBLS3637). All data supporting the findings of this study are available in the manuscript or the supplementary information. Source data are provided with this paper.

Field-specific reporting

Please select the one below that is the best fit for your research. If you are not sure, read the appropriate sections before making your selection.

☒ Life sciences ☐ Behavioural & social sciences ☐ Ecological, evolutionary & environmental sciences

For a reference copy of the document with all sections, see nature.com/documents/nr-reporting-summary-flat.pdf

Life sciences study design

All studies must disclose on these points even when the disclosure is negative.

Sample size	Sample sizes were chosen based on those used in similar studies to which we expected similar effect sizes in our current experiments (Li et al., 2017, Proc. R. Soc. B Biol. Sci.; Schretter et al., 2018, Nature).
Data exclusions	No data were excluded.
Replication	For all experiments, more than four biological replicates were used, as noted in the manuscript, to yield reproducible results. All attempts at replication were successful.
Randomization	Bees were randomly assigned to different treatment groups.
Blinding	Experimenters were not blinded to group allocation, which is not practical as different groups needed specific treatments (e.g., with or without L. apis administration). All data analyses were performed automatically with the same parameter setting for each experiment.

Reporting for specific materials, systems and methods

We require information from authors about some types of materials, experimental systems and methods used in many studies. Here, indicate whether each material, system or method listed is relevant to your study. If you are not sure if a list item applies to your research, read the appropriate section before selecting a response.

Materials & experimental systems

n/a	Involved in the study	
☒	☐	Antibodies
☒	☐	Eukaryotic cell lines
☒	☐	Palaeontology and archaeology
☐	☒	Animals and other organisms
☒	☐	Human research participants
☒	☐	Clinical data
☒	☐	Dual use research of concern

Methods

n/a	Involved in the study	
☒	☐	ChIP-seq
☒	☐	Flow cytometry
☒	☐	MRI-based neuroimaging

Animals and other organisms

Policy information about studies involving animals; ARRIVE guidelines recommended for reporting animal research

Laboratory animals	Worker bumblebees (Bombus terrestris) were used in this study. All bees had similar age (12.18 ± 0.52 days) at time of collection.
Wild animals	The study did not involve wild animals.
Field-collected samples	The study did not involve field-collected samples.
Ethics oversight	No ethical guidance was required for the use of bumblebees.

Note that full information on the approval of the study protocol must also be provided in the manuscript.
